# Surgery formulae for the Seiberg–Witten invariant of plumbed 3-manifolds

**DOI:** 10.1007/s13163-019-00297-z

**Published:** 2019-05-05

**Authors:** Tamás László, János Nagy, András Némethi

**Affiliations:** 1 BCAM - Basque Center for Applied Mathematics, Mazarredo, 14, E48009 Bilbao, Basque Country Spain; 2 Department of Mathematics, Central European University, Budapest, Hungary; 3 Present Address: Alfréd Rényi Institute of Mathematics, Hungarian Academy of Sciences, Reáltanoda utca 13-15, Budapest, 1053 Hungary; 4 Department of Geometry, ELTE - University of Budapest, Budapest, Hungary; 5 Alfréd Rényi Institute of Mathematics, Hungarian Academy of Sciences, Reáltanoda utca 13-15, Budapest, 1053 Hungary

**Keywords:** Normal surface singularities, Seiberg–Witten invariant, Plumbing graphs, Rational homology spheres, Poincaré series, Quasipolynomials, Surgery formula, Periodic constant, MSC 32S05, MSC 32S25, MSC 32S50, MSC 57M27

## Abstract

Assume that $M({{\mathcal {T}}})$ is a rational homology sphere plumbed 3-manifold associated with a connected negative definite graph $\mathcal {T}$. We consider the combinatorial multivariable Poincaré series associated with $\mathcal {T}$ and its counting functions, which encode rich topological information. Using the ‘periodic constant’ of the series (with reduced variables associated with an arbitrary subset ${{\mathcal {I}}}$ of the set of vertices) we prove surgery formulae for the normalized Seiberg–Witten invariants: the periodic constant associated with ${{\mathcal {I}}}$ appears as the difference of the Seiberg–Witten invariants of $M({{\mathcal {T}}})$ and $M({{\mathcal {T}}}{\setminus }{{\mathcal {I}}})$ for any ${{\mathcal {I}}}$.

## Introduction

Surgery formulae for 3-manifolds, focusing on certain numerical or cohomological invariant are key tools in low dimensional topology. They can serve e.g. in the identification of invariants, or in the proof of the coincidence of two differently defined one, but also in concrete computations of the invariants for certain families of manifolds. Some numerical surgery formulae are consequences of cohomological exact sequences, where the involved cohomological theories are categorifications of the corresponding numerical invariants. E.g., as the Seiberg–Witten invariant admits several categorifications—the Heegaard Floer homology of Ozsváth and Szabó, or the monopole homology of Kronheimer and Mrowka, or (in the case of plumbed manifolds) the lattice cohomology introduced by the third author—, exact sequences in these theories induce surgery formulae for the Seiberg–Witten invariant as well, see e.g. [11, 26, 35]. Usually, such exact triangles compare the invariants of three surgery 3–manifolds, see again [35].

However, for negative definite graph manifolds, one can formulate a different type of surgery formula, which is not imposed by purely topological theories and it has no extension (by the knowledge of the authors) to arbitrary 3-manifolds. It has its roots in complex algebraic/analytic geometry by surgery formulae associated with analytic invariants, where certain Hilbert series play crucial role, see e.g. [34]. Using the fact that negative definite graph manifolds are exactly the links of normal surface singularities, one can try to transport such ideas from the analytic theory giving rise to purely topological results. By the new formula we present, the difference of the Seiberg–Witten invariants of two surgery manifolds is determined from a multivariable zeta–type series, which is combinatorially defined from the graph. This series is the topological analogue of a Poincaré series of a multivariable divisorial filtration (of a local analytic algebra), but in this topological/combinatorial discussion the analytic part can be totally neglected (however, for such connections see [5, 20–23, 25, 28]).

In the sequel $M({{\mathcal {T}}})$ denotes a plumbed 3-manifold associated with a connected negative definite graphs ${{\mathcal {T}}}$. We will assume that $M({{\mathcal {T}}})$ is a rational homology sphere (hence ${{\mathcal {T}}}$ is a tree of $S^2$’s).

The series $Z({{\mathbf {t}}})$, which guides several topological invariants of $M({{\mathcal {T}}})$, is defined combinatorially from ${{\mathcal {T}}}$, see (1). The number of variables $\{t_v\}_v$ is indexed by the set of vertices ${{\mathcal {V}}}$ of ${{\mathcal {T}}}$. The series $Z({{\mathbf {t}}})$ decomposes as a sum $Z_h({{\mathbf {t}}})$ according to the $\hbox {spin}^c$–structures of $M({{\mathcal {T}}})$.

If $S({{\mathbf {t}}})=\sum _{l'}s(l'){{\mathbf {t}}}^{l'}$ is a multivariable series, then its counting function $Q(l')$ is defined by $Q(l'_0)=\sum _{l'\not \ge l'_0} s(l')$. We say that *S* admits a quasipolynomial (in the cone ${{\mathcal {K}}}$) if for elements $l'_0$ from a shifted cone of type $l^*+{{\mathcal {K}}}$ the value $Q(l'_0)$ equals the value $\mathfrak {Q}(l'_0)$ of a quasipolynomial $\mathfrak {Q}$ (for precise definitions see 2.4). In this case we define the *periodic constant * of *S* (associated with ${{\mathcal {K}}}$) as $\mathfrak {Q}(0)$. The construction creates a bridge between topological invariants of $M({{\mathcal {T}}})$ and generalized Ehrhart theory of counting functions and their quasipolynomials (see e.g. [14]). The above construction applied for $Z_h$ realizes a deep connection between low dimensional topology and multivariable (Poincaré type) series and their periodic constants. Indeed, by [25], the periodic constant of $Z_h$ (associated with the Lipman cone of ${{\mathcal {T}}}$) equals the normalized Seiberg–Witten invariant of *M* (where *h* indexes the corresponding $\hbox {spin}^c$–structures). For the precise statement see Theorems 1 and 2.

The surgery formula which (partly) motivated our research is the following [5]. Let us fix a vertex $v\in {{\mathcal {V}}}$ and consider the graph ${{\mathcal {T}}}{\setminus }v$ obtained from ${{\mathcal {T}}}$ by eliminating *v*. Then the difference of the normalized Seiberg–Witten invariants of $M({{\mathcal {T}}})$ and $M({{\mathcal {T}}}{\setminus }v)$ can be computed as the periodic constant of the one–variable series $Z_h({{\mathbf {t}}})|_{t_u=1, \, u\not =v}$ (cf. Theorem 4).

One of the main results of the present work (see Theorem 5) is a common generalization of the above results. We fix an arbitrary subset ${{\mathcal {I}}}\subset {{\mathcal {V}}}$ of the vertices of ${{\mathcal {T}}}$, and we prove that the difference of the normalized Seiberg–Witten invariants of $M({{\mathcal {T}}})$ and $M({{\mathcal {T}}}{\setminus }{{\mathcal {I}}})$ can be computed as the periodic constant of the series with reduced variables $Z_h({{\mathbf {t}}})|_{t_u=1, \, u\not \in {{\mathcal {I}}}}$. (Note that the theory of quasipolynomials and also the concrete computation of their periodic constants is much harder in the multivariable case.) In the case when ${{\mathcal {I}}}$ is the set of nodes of ${{\mathcal {T}}}$ then we recover the ‘reduction theorem’ from [15]. In the cases when ${{\mathcal {T}}}{\setminus }{{\mathcal {I}}}$ contains only strings or ‘rational graphs’ (that is, $M({{\mathcal {T}}}{\setminus }{{\mathcal {I}}})$ is an L–space) then the formula simplifies, and we get a closed formula of the normalized Seiberg–Witten invariants of $M({{\mathcal {T}}})$ in terms of $Z_h$ with reduced variables.

It is important to mention that the ‘classical’ exact triangles (like in [35]), hence their surgery formulae too, involve manifolds which are modified along a knot. This, in the language of plumbing graph means modification along one of the vertices. On the other hand, our formula is more general, since ${{\mathcal {I}}}$ can be an arbitrary subset of vertices. Additionally, our formulae separates the involved $\hbox {spin}^c$–structures (while exact triangles usually mix them).

The organization of the paper is the following. Sect. 2 contains preliminaries regarding plumbing graphs, manifolds, their Seiberg–Witten invariants, and also Poincaré series and their periodic constants. We also recall several key Seiberg–Witten invariant formulae which will be used and generalized later.

In Sect. 3 we formulate the new results and we list several applications. We split the presentation into two steps: we give a ‘numerical surgery formula’ (Theorem 5) targeting the Seiberg–Witten invariant, and also another surgery formula, which is a lift of the numerical identity to the level of quasipolynomials (Theorem 6).

In Sects. 4, 5 and 6 we prove the new results. In the proof we decompose the counting function into an alternating sum of ‘modified counting functions’. In Sect. 4 prove a ‘convexity property’ of these sums, in Sect. 5 a surgery formula for them, and finally in Sect. 6 we finish the proof.

Section 7 treats the case when ${{\mathcal {I}}}$ is the set of nodes (hence ${{\mathcal {T}}}{\setminus }{{\mathcal {I}}}$ are strings), while Sect. 8 the case when all subgraphs ${{\mathcal {T}}}{\setminus }{{\mathcal {I}}}$ are rational. In these cases several vanishing results are established. Here several computations are based on the (positive answer to the) Seiberg–Witten Invariant Conjecture from [5, 22, 27] and we also explain (and use) the connections with the analytic (singularity theoretical) counterpart as well.

The last section treats the case of numerically Gorenstein graphs, where some additional nice symmetries and dualities appear.

## Preliminaries

For more details regarding plumbing graphs, plumbed manifolds and their relations with normal surface singularities see [5, 9, 20–23, 25, 28, 31]; for Poincaré series see also [6–8].

### Plumbing graphs: plumbed 3-manifolds

We fix a connected plumbing graph $\mathcal {T}$ whose associated intersection matrix is negative definite. We denote the corresponding plumbed 3-manifold by $M=M({{\mathcal {T}}})$. In this article we always assume that *M* is an oriented rational homology sphere, equivalently, $\mathcal {T}$ is a tree with all genus decorations zero.

We use the notation $\mathcal {V}$ for the set of vertices, $\delta _v$ for the valency of a vertex *v*, and $\mathcal {N}$ for the set of nodes, i.e. vertices with $\delta _v\ge 3$. End–vertices are defined by $\delta _v=1$.

Let $\widetilde{X}$ be the plumbed 4–manifold with boundary associated with $\mathcal {T}$, hence $\partial \widetilde{X} = M$. Its second homology $L:=H_2(\widetilde{X},\mathbb {Z})$ is a lattice, freely generated by the classes of 2–spheres $\{E_v\}_{v\in \mathcal {V}}$, with a negative definite intersection form $(\,,\,)$. Furthermore, $H^2(\widetilde{X},\mathbb {Z})$ can be identified with the dual lattice $L':=\mathrm{Hom}_{\mathbb {Z}}(L,{\mathbb {Z}})=\{l'\in L\otimes {\mathbb {Q}}\,:\, (l',L)\in {\mathbb {Z}}\}$. It is generated by the (anti)dual classes $\{E^*_v\}_{v\in \mathcal {V}}$ defined by $(E^{*}_{v},E_{w})=-\delta _{vw}$, the opposite of the Kronecker symbol. One has the inclusions $L\subset L'\subset L\otimes {\mathbb {Q}}$, and $H_1(M,\mathbb {Z})\simeq L'/L$, denoted by *H*. We write [*x*] for the class of $x\in L'$ in *H*.

For any $h\in H$ let $r_h\in L'$ be its unique representative in the ‘semi–open cube’ $\{\sum _vl'_vE_v\in L'\,:\, l'_v\in [0,1)\}$.

$L'$ carries a partial ordering induced by $l'=\sum _vl'_vE_v\ge 0$ if and only if each $l'_v\ge 0$.

### The series $Z({\mathbf {t}})$

The *multivariable topological Poincaré series* is the Taylor expansion $Z({\mathbf {t}})=\sum _{l'} z^{{\mathcal {T}}}(l'){{\mathbf {t}}}^{l'} \in {\mathbb {Z}}[[L']]$ at the origin of the rational function

1 $$\begin{aligned} f({\mathbf {t}})=\prod _{v\in \mathcal {V}} (1-{\mathbf {t}}^{E^*_v})^{\delta _v-2}, \end{aligned}$$

where ${{\mathbf {t}}}^{l'}:=\prod _{v\in \mathcal {V}}t_v^{l'_v}$ for any $l'=\sum _{v\in \mathcal {V}}l'_vE_v\in L'$ ($l'_v\in {\mathbb {Q}}$). It decomposes as $Z({\mathbf {t}})=\sum _{h\in H}Z_h({\mathbf {t}})$, where $Z_h({\mathbf {t}})=\sum _{[l']=h}z^{{\mathcal {T}}}(l'){{\mathbf {t}}}^{l'}$. The expression (1) shows that $Z({\mathbf {t}})$ is supported in the *Lipman cone*$\mathcal {S}':=\mathbb {Z}_{\ge 0}\langle E^{*}_{v}\rangle _{v\in \mathcal {V}}$. Since *I* is negative definite, all the entries of $E_v^*$ are strictly positive, hence ${{\mathcal {S}}}'\subset \{\sum _vl'_vE_v\,:\, l'_v>0\}\cup \{0\}$. Thus, for any *x*, $\{l'\in {{\mathcal {S}}}'\,:\, l'\not \ge x\}$ is finite, cf. [25, (2.1.2)].

Fix $h\in H$. We define a ‘counting function’ of the coefficients of $Z_h$ by

2 $$\begin{aligned} Q^{{\mathcal {T}}}_h: L'_h:=\{x \in L'\,:\, [x]=h\}\rightarrow {\mathbb {Z}}, \ \ \ \ Q^{{\mathcal {T}}}_{h}(x)=\sum _{l'\ngeq x,\, [l']=h} z^{{\mathcal {T}}}(l'). \end{aligned}$$

For the motivation of the truncation $\{l'\ngeq x,\, [l']=h\}$ see the results below (e.g. 3) or [27].

### Seiberg–Witten invariants of *M*

The 4–manifold $\widetilde{X}$ has a complex structure. In fact, any such $M({{\mathcal {T}}})$ is the link of a complex normal surface singularity (*X*, *o*), which has a resolution $\widetilde{X}\rightarrow X$ with resolution graph ${{\mathcal {T}}}$ (see e.g. [20]). In this analytic case $\widetilde{X}$ is a smooth complex 2–manifold, and as a real smooth manifold is the disc–plumbed 4-manifold associated with ${{\mathcal {T}}}$. Let $K\in L'$ be its canonical cycle. Though the complex structure (with fixed ${{\mathcal {T}}}$) is not unique, *K* is determined topologically by *L* via the adjunction formulae $(K+E_v,E_v)+2=0$ for all *v*. Let ${\widetilde{\sigma }}_{can}$ be the *canonical*$spin^c$*—structure on*$\widetilde{X}$ identified by $c_1({\widetilde{\sigma }}_{can})=-K$, and let $\sigma _{can}\in \mathrm {Spin}^c(M)$ be its restriction to *M*, called the *canonical*$spin^c$–*structure onM* (cf. [10]). $\mathrm {Spin}^c(M)$ is an *H*–torsor with action denoted by $*$.

We denote by $\mathfrak {sw}_{\sigma }(M)\in {\mathbb {Q}}$ the *Seiberg–Witten invariant* of *M* indexed by the $spin^c$–structures $\sigma \in \mathrm{Spin}^c(M)$ (cf. [17, 32]). (We will use the sign convention of [5, 25].)

In the last years several combinatorial expressions were established for the Seiberg–Witten invariants. For rational homology spheres, Nicolaescu [32] showed that $\mathfrak {sw}(M)$ is equal to the Reidemeister–Turaev torsion normalized by the Casson–Walker invariant. In the case when *M* is a negative definite plumbed rational homology sphere, a combinatorial formula for Casson–Walker invariant in terms of the plumbing graph can be found in Lescop [19], and the Reidemeister–Turaev torsion is determined by Némethi and Nicolaescu [28] using Dedekind–Fourier sums.

A different combinatorial formula of $\{\mathfrak {sw}_\sigma (M)\}_\sigma$ was proved in [25] using qualitative properties of the coefficients of the series $Z({\mathbf {t}})$.

#### Theorem 1

[25] For any $$l'\in -K+ int (\mathcal {S}')$$

3 $$\begin{aligned} Q_{[l']}(l')= -\mathfrak {sw}_{[-l']*\sigma _{can}}(M)-\frac{(K+2l')^2+|\mathcal {V}|}{8}. \end{aligned}$$

If we fix $h\in H$ and we write $l'=l+r_{h}$ with $l\in L$, then the right hand side of (3) is a multivariable quadratic polynomial on *L*, a fact which will be exploited conceptually next.

### Periodic constants

A key tool of the present article is an invariant associated with series motivated by properties of Hilbert–Samuel functions used in algebraic geometry and singularity theory. This also creates a bridge with Ehrhart theory and the properties of its qusipolynomials. It is called the *periodic constant* of the series. For one–variable series they were introduced in [29, 34], see also [5], the multivariable generalization is treated in [14].

Let $S(t)=\sum _{l\ge 0}c_l t^l \in \mathbb {Z}[[t]]$ be a formal power series with one variable. Assume that for some $p\in {\mathbb {Z}}_{>0}$ the counting function $Q^{(p)}(n):=\sum _{l=0}^{pn-1}c_l$ is a polynomial ${\mathfrak {Q}}^{(p)}$ in *n*. Then the constant term ${\mathfrak {Q}}^{(p)}(0)$ is independent of *p* and it is called the *periodic constant*$\mathrm {pc}(S)$ of the series *S*. E.g., if *S*(*t*) is a finite polynomial, then $\mathrm {pc}(S)$ exists and it equals *S*(1). If the coefficients of *S*(*t*) are given by a Hilbert function $l\mapsto c(l)$, which admits a Hilbert polynomial *H*(*l*) with $c(l)=H(l)$ for $l\gg 0$, then $S^{reg}(t)=\sum _{l\ge 0}H(l)t^l$ has zero periodic constant and $\mathrm {pc}(S)=\mathrm {pc}(S-S^{reg})+\mathrm {pc}(S^{reg})=(S-S^{reg})(1)$, measuring the difference between the Hilbert function and Hilbert polynomial.

For the multivariable case we consider a (negative) definite lattice $L=\mathbb {Z}\langle E_v\rangle _v$, its dual lattice $L'$, a series $S({\mathbf {t}})\in {\mathbb {Z}}[[L']]$ (e.g. $Z({{\mathbf {t}}})$), and its well-defined counting function $Q_h=Q_h(S({{\mathbf {t}}}))$ as in (2) for fixed $h\in L'/L$. Assume that there exist a real cone $\mathcal {K}\subset L'\otimes \mathbb {R}$ whose affine closure is top–dimensional, $l'_* \in \mathcal {K}$, a sublattice $\widetilde{L} \subset L$ of finite index, and a quasipolynomial $\mathfrak {Q}_h(l)$ ($l\in \widetilde{L}$) such that $Q_h(l+r_h)=\mathfrak {Q}_h(l)$ for any $l+r_h\in (l'_* +\mathcal {K})\cap (\widetilde{L}+r_h)$. Then we say that the counting function $Q_h$ (or just $S_h({\mathbf {t}})$) admits a quasipolynomial in $\mathcal {K}$, namely $\mathfrak {Q}_h(l)$, and also an (equivariant, multivariable) *periodic constant* associated with $\mathcal {K}$, which is defined by

4 $$\begin{aligned} \mathrm {pc}^{\mathcal {K}}(S_h({{\mathbf {t}}})) :=\mathfrak {Q}_h(0). \end{aligned}$$

The definition does not depend on the choice of the sublattice ${\widetilde{L}}$, which corresponds to the choice of *p* in the one–variable case. This is responsible for the name ‘periodic’ in the definition. The definition is independent of the choice of $l'_*$ as well.

By general theory of multivariable Ehrhart-type quasipolynomials (counting special coefficients of lattice points in polytopes attached to $Z({\mathbf {t}})$) one can construct a conical chamber decomposition of the space $L'\otimes \mathbb {R}$, such that each cone satisfies the above definition (hence provides a periodic constant), for details see [14] or [38]. This decomposition, in principle, divides $\mathcal {S}'_{\mathbb {R}} :=\mathcal {S}'\otimes \mathbb {R}$ into several sub–cones (hence, providing different quasipolynomials and periodic constants associated with these sub–cones of $\mathcal {S}'_{\mathbb {R}}$). However, Theorem 1 guarantees that this is not the case, the whole $\mathcal {S}'_{\mathbb {R}}$ is a unique chamber (cf. also with [16]). Hence, Theorem 1 reads as follows.

#### Theorem 2

[25] The counting function of $Z_h({{\mathbf {t}}})$ in the cone $S'_{\mathbb {R}}$ admits the (quasi)polynomial

5 $$\begin{aligned} \mathfrak {Q}_{h}(l)= -\mathfrak {sw}_{-h*\sigma _{can}}(M)-\frac{(K+2r_h+2l)^2+|\mathcal {V}|}{8}, \end{aligned}$$

whose periodic constant is

6 $$\begin{aligned} \mathrm {pc}^{S'_{\mathbb {R}}}(Z_h({\mathbf {t}}))=\mathfrak {Q}_h(0)= -\mathfrak {sw}_{-h*\sigma _{can}}(M)-\frac{(K+2r_h)^2+|\mathcal {V}|}{8}. \end{aligned}$$

The right hand side of (6) with opposite sign is called the $r_h$-*normalized Seiberg–Witten invariant* of *M*.

### Reduced Poincaré series

Fix $h\in H$. For any ${{\mathcal {I}}}\subset {{\mathcal {V}}}$, ${{\mathcal {I}}}\not =\emptyset$, we define the *reduced rational function*

$$\begin{aligned} f_h({\mathbf {t}}_{{{\mathcal {I}}}}):=f_h({\mathbf {t}})|_{t_v=1,v\notin {{\mathcal {I}}}} \end{aligned}$$

and its Taylor expansion $Z_h({\mathbf {t}}_{{{\mathcal {I}}}})$, called the *reduced Poincaré series*. Note that $Z_h({\mathbf {t}}_{{{\mathcal {I}}}})$ can be obtained as $Z_h({\mathbf {t}})|_{t_v=1,v\notin {{\mathcal {I}}}}$ as well (this is well defined: the summations of the corresponding coefficients are *finite*, since $Z({{\mathbf {t}}})$ is supported on ${{\mathcal {S}}}'$). Also, it is important to notice that before the elimination of certain variables, we have to decompose the series $Z({\mathbf {t}})=\sum _{h}Z_h({\mathbf {t}})$ into its components $Z_h({{\mathbf {t}}})$, since the reduced (total) series $Z({{\mathbf {t}}}_{{{\mathcal {I}}}})$ does not contain sufficient information, which might provide the decomposition into its components $\{Z_h({{\mathbf {t}}}_{{\mathcal {I}}})\}_h$.

For any such ${{\mathcal {I}}}$, one defines several operators connecting the different lattices. First, we define the projection (along the *E*–coordinates) $\pi _{{{\mathcal {I}}}}:\mathbb {R}\langle E_v\rangle _{v\in \mathcal {V}}\rightarrow \mathbb {R}\langle E_v\rangle _{v\in {{\mathcal {I}}}}$, denoted also as $x\mapsto x|_{{{\mathcal {I}}}}$, by $\sum _{v\in {{\mathcal {V}}}}l_vE_v\mapsto \sum _{v\in {{\mathcal {I}}}}l_vE_v$. Note that if ${{\mathcal {I}}}$ is identified with the set of vertices of a subgraph ${{\mathcal {T}}}_{{\mathcal {I}}}$, then $\pi _{{\mathcal {I}}}$ does not preserve the intersection form in the corresponding lattices $L({{\mathcal {T}}})$ and $L({{\mathcal {T}}}_{{\mathcal {I}}})$.

$\pi _{{\mathcal {I}}}$ provides the ‘projected (real) Lipman cone’ $\pi _{{{\mathcal {I}}}}(S'_{\mathbb {R}})$.

We wish to understand what happens with the information coded in $Z_h$ after elimination certain variables. The next results, as a prototype, shows that under certain reduction the ‘Seiberg–Witten information’ survives: if the set of nodes $\mathcal {N}$ is non–empty then for ${{\mathcal {I}}}=\mathcal {N}$ one has the following.

#### Theorem 3

[14] The counting function of $Z_h({{\mathbf {t}}}_{\mathcal {N}})$ in the cone $\pi _{\mathcal {N}}(S'_{\mathbb {R}})$ admits a quasipolynomial and a periodic constant, and

$$\begin{aligned} \mathrm {pc}^{\pi _{\mathcal {N}}(S'_{\mathbb {R}})}(Z_h({\mathbf {t}}_{\mathcal {N}}))=\mathrm {pc}^{S'_{\mathbb {R}}}(Z_h({\mathbf {t}}))= -\mathfrak {sw}_{-h*\sigma _{can}}(M)-\frac{(K+2r_h)^2+|\mathcal {V}|}{8}. \end{aligned}$$

This result has the following advantages: the number of reduced variables (i.e. number of nodes) usually is considerably less than the number of vertices, a fact which reduces the complexity of the calculations. Moreover, the reduced series reflects more conceptually the complexity of the manifold *M* (using only one variable for each Seifert 3-manifold piece in its JSJ–decomposition). Furthermore, the reduced series can be compared/linked with other (geometrically or analytically defined) objects as well (see e.g. [5, 23]).

We generalize this result in two directions: first, we replace $\mathcal {N}$ by an arbitrary subset ${{\mathcal {I}}}\not =\emptyset$, and second, we lift the identity from the numerical periodic constant level to the quasipolynomial level.

### A surgery formula associated with the elimination of a vertex

Surgery formulae for a certain 3-manifold invariant, in general, compare the invariant of *M* with the invariants of different surgery modifications of *M*. In the case of plumbed 3-manifolds, one compares the invariants associated with 3-manifolds obtained by different modifications of the graph. The ‘standard’ topological surgery formulae for the Seiberg–Witten invariant (induced by exact triangles of certain cohomology theories, cf. [11, 26, 35]) compare the invariants of three such 3-manifolds. Furthermore, in these approaches, one cannot separate a certain fixed $spin^c$ structure, the theory mixes always several of them. (See also [39].) The next formula (and our generalizations as well) are different: they compare the Seiberg–Witten invariant of two 3-manifolds via an ‘algebraic’ term defined as the periodic constant of a series (and they split according to the $spin^c$–structures).

Let us fix ${{\mathcal {I}}}\subset {{\mathcal {V}}}$. The vertices of the connected components of ${{\mathcal {V}}}{\setminus }{{\mathcal {I}}}$ together with the edges connecting them in $\mathcal {T}$ determine the connected full subgraphs $\{{{\mathcal {T}}}_i\}_i$, $\cup _i{{\mathcal {T}}}_i={{\mathcal {T}}}{\setminus }{{\mathcal {I}}}$. For each *i* we consider the inclusion operator $j_i:L({{\mathcal {T}}}_i)\rightarrow L({{\mathcal {T}}})$, $E_v({{\mathcal {T}}}_i)\mapsto E_v({{\mathcal {T}}})$, identifying naturally the corresponding *E*–base elements in the two graphs. This preserves the intersection forms. Let $j_{i}^*: L'({{\mathcal {T}}})\rightarrow L'({{\mathcal {T}}}_i)$ be its dual, defined by $j_{i}^*(E^*_{v}({{\mathcal {T}}}))=E^*_{v}({{\mathcal {T}}}_i)$ if $v\in {{\mathcal {V}}}({{\mathcal {T}}}_i)$, and $j_{i}^*(E^*_{v}({{\mathcal {T}}}))=0$ otherwise. Then $(j^*_{i}(l'), l)_{{{\mathcal {T}}}_i}=(l',j_{i}(l))_{{{\mathcal {T}}}}$ for any $l'\in L'({{\mathcal {T}}})$ and $l\in L({{\mathcal {T}}}_i)$.

Let us start with an arbitrary $spin^c$–structure ${\widetilde{\sigma }}$ on $\widetilde{X}$. Since $\mathrm{Spin}^c(\widetilde{X})$ is an $L'$–torsor, there is a unique $l'\in L'$ such that ${\widetilde{\sigma }}=l'*{\widetilde{\sigma }}_{can}$. Its restriction to $\mathrm{Spin}^c(M)$ is $\sigma =[l']*\sigma _{can}$. We also refer to ${\widetilde{\sigma }}$ as the extension of $\sigma$. Since $\widetilde{X}({{\mathcal {T}}}_i)$ can be regarded as a small tubular neighbourhood of those $E_v$ which are contained in ${{\mathcal {V}}}({{\mathcal {T}}}_i)$, ${\widetilde{\sigma }}$ has restrictions ${\widetilde{\sigma }}_i$ to each $\widetilde{X}({{\mathcal {T}}}_i)$ too. Since the canonical $spin^c$–structure of ${\widetilde{X}}$ restricts to the canonical $spin^c$–structure ${{\widetilde{\sigma }}}_{can,i}$ of $\widetilde{X}({{\mathcal {T}}}_i)$, ${{\widetilde{\sigma }}}=l'*{\widetilde{\sigma }}_{can}$ restricts to ${{\widetilde{\sigma }}}_i:=j_{i}^*(l')*{{\widetilde{\sigma }}}_{can,i} \in \mathrm{Spin}^c(\widetilde{X}({{\mathcal {T}}}_i))$, whose restriction to the boundary $M_i=M({{\mathcal {T}}}_i)=\partial \widetilde{X}_i$ is $\sigma _i=[j_{i}^*(l')]*\sigma _{can,i}$.

Having these general definitions, let us consider first the particular case of ${{\mathcal {I}}}=\{v\}$ ($v\in {{\mathcal {V}}}$), and, as above, let $\{{{\mathcal {T}}}_i\}_i$ be the connected components of ${{\mathcal {T}}}\setminus \{v\}$. The following surgery formula was one of the motivations of our main result. Below the reduced ${{\mathbf {t}}}_{{\mathcal {I}}}$ has only one variable, namely $t_v$, and the corresponding periodic constant is computed by the ‘easy’ definition of the one–variable series.

#### Theorem 4

[5] Fix any $h\in H$ and extend $h*\sigma _{can}\in \mathrm{Spin}^c(M)$ as ${\widetilde{\sigma }}:= r_h*{\widetilde{\sigma }}_{can}\in \mathrm{Spin}^c(\widetilde{X})$. Consider also ${{\mathcal {I}}}=\{v\}\subset {{\mathcal {V}}}$ and the corresponding restrictions of ${\widetilde{\sigma }}$ to $\cup _i M({{\mathcal {T}}}_i)$. Then the series $Z_h(t_{{\mathcal {I}}})=Z_h(t_v)$ admits a periodic constant, and

$$\begin{aligned} \begin{aligned}&\mathfrak {sw}_{-h*\sigma _{can}}(M)+\frac{(K+2r_h)^2+|\mathcal {V}|}{8}\\&\quad =\ \sum _{i}\, \Big ( \mathfrak {sw}_{-[j^*_{i}(r_h)]*\sigma _{can,i}}(M_i)+\frac{(K(\mathcal {T}_i) + 2j^*_i(r_h))^2+ |\mathcal {V}({{\mathcal {T}}}_i))|}{8}\Big )\\&\qquad - pc (Z_{h}(t_{v})). \end{aligned} \end{aligned}$$

(Note that usually $j^*_i(r_h)\not = r_{[j^*_i(r_h)]}$, see below.) This will be generalized to arbitrary ${{\mathcal {I}}}\not =\emptyset$ and to an arbitrary extension ${\widetilde{\sigma }}:= l'*{\widetilde{\sigma }}_{can}$ ($l'\in L'$) of $h*\sigma _{can}$.

## The main result: the new surgery formulae

### Surgery formula for the Seiberg–Witten invariant

First we state a consequence of our Main Theorem 6, which is still sufficiently general to generalize all the previous results.

We will use the notations of the previous section. Let ${{\mathcal {I}}}\subset {{\mathcal {V}}}$ be an arbitrary non–empty subset and write ${{\mathcal {T}}}\setminus {{\mathcal {I}}}$ as the union of full connected subgraphs $\cup _i{{\mathcal {T}}}_i$. Moreover, we fix $h\in H$ as well.

#### Theorem 5

The series $Z_{h}({\mathbf {t}}_{{\mathcal {I}}})$ admits a periodic constant in the real cone $\pi _{{\mathcal {I}}}({{\mathcal {S}}}'_{\mathbb {R}})$, and

$$\begin{aligned} \begin{aligned}&\mathfrak {sw}_{-h*\sigma _{can}}(M)+\frac{(K+2r_h)^2+|\mathcal {V}|}{8}\\&\quad =\ \sum _{i} \Big ( \mathfrak {sw}_{-[j^*_{i}(r_h)]*\sigma _{can,i}}(M_i)+\frac{(K(\mathcal {T}_i) + 2j^*_i(r_h))^2+ |\mathcal {V}({{\mathcal {T}}}_i)|}{8}\Big )\\&\qquad - pc ^{\pi _{{\mathcal {I}}}({{\mathcal {S}}}'_{\mathbb {R}})}(Z_{h}({\mathbf {t}}_{{\mathcal {I}}})). \end{aligned} \end{aligned}$$

#### Corollary 1

Let us consider the following particular cases.

1. Assume that ${{\mathcal {I}}}={{\mathcal {V}}}$. Then each ${{\mathcal {T}}}_i$ is empty, and we recover Theorem 2 proved in [25].
2. Assume that ${{\mathcal {I}}}$ consists of one vertex. Then we recover Theorem 4 proved in [5].
3. Assume that ${{\mathcal {I}}}=\mathcal {N}$. Then we recover Theorem 3, proved in [14], once we verify for each *i* the vanishing
  7 $$\begin{aligned} \mathfrak {sw}_{-[j^*_{i}(r_h)]*\sigma _{can,i}}(M_i)+\frac{(K(\mathcal {T}_i) + 2j^*_i(r_h))^2+ |\mathcal {V}({{\mathcal {T}}}_i)|}{8}=0. \end{aligned}$$
  This vanishing is well–known for $h=0$ (see e.g.  8.2 or Remark 4), but it is not evident at all (at least for the authors) for arbitrary *h*. It will be proved in Sect. 7 by analytic methods. In this case the graph ${{\mathcal {T}}}_i$ is a string, hence $M({{\mathcal {T}}}_i)$ is a lens space. The difficulty in the vanishing (7) is that $r_h$ is a global object induced from ${{\mathcal {T}}}$, and for any fixed ${{\mathcal {T}}}_i$ is not clear at all what classes of $L'({{\mathcal {T}}}_i)$ might appear as $j^*_i(r_h)$ for a certain bigger graph ${{\mathcal {T}}}\supset {{\mathcal {T}}}_i$. (If $h=0$ then $j^*_i(r_h)=0$ as well, which simplifies the situation: (7) is a statement regarding merely a lens space, and it follows e.g. from (37).)
4. For generalization of (3) for the case when all subgraphs ${{\mathcal {T}}}_i$ are *rational*, see Sect. 8.

### Surgery formula for the counting function

The above formula from Theorem 5, which targets numerical invariants, is a consequence of a general ‘*surgery identity of quasipolynomials*’. This is the subject of the Main Theorem 6.

Similarly to the counting functions defined in (2), we set for any *h* and ${{\mathcal {I}}}\subset {{\mathcal {V}}}$, ${{\mathcal {I}}}\not =\emptyset$,

8 $$\begin{aligned} Q^{{\mathcal {T}}}_{h,{{\mathcal {I}}}}: L'_{h}\rightarrow {\mathbb {Z}}, \ \ \ \ Q^{{\mathcal {T}}}_{h,{{\mathcal {I}}}}(x):=\sum _{l'|_{{\mathcal {I}}}\ngeq x |_{{\mathcal {I}}}, \, [l']=[x]} z^{{\mathcal {T}}}(l'). \end{aligned}$$

Note that $Q^{{\mathcal {T}}}_{h,{{\mathcal {I}}}}$ depends only on the reduced series $Z_h({{\mathbf {t}}}_{{\mathcal {I}}})$: it is its counting function.

The setup of the next statement is the following. We fix $h\in H$, and we choose $l'_0=\sum _{v\in {{\mathcal {V}}}} a_vE^*_v\in L'$ with

$[l'_0]=h$. We also fix ${{\mathcal {I}}}\subset {{\mathcal {V}}}$, ${{\mathcal {I}}}\not =\emptyset$, and ${{\mathcal {T}}}{\setminus }{{\mathcal {I}}}=\cup _i{{\mathcal {T}}}_i$.

#### Theorem 6

For any $l'_0$ with all $a_v$ sufficiently large one has the identity

9 $$\begin{aligned} Q^{{\mathcal {T}}}_{[l'_0]}\,(l'_0)=Q^{{\mathcal {T}}}_{[l'_0],{{\mathcal {I}}}}\,(l'_0)+\sum _i \ Q^{{{\mathcal {T}}}_i}_{[j^*_i(l'_0)]}(j^*_i(l'_0)). \end{aligned}$$

### Consequences and corollaries

Let us deduce some consequences and corollaries.

Write $l'_0=r_h+l$. By Theorem 1 (see Theorem 2 too), for all $a_v$ large, $Q^{{\mathcal {T}}}_{[l'_0]}(l'_0)$ equals the (quasi)polynomial $\mathfrak {Q}^{{\mathcal {T}}}_h(l)$, and the same is true for each term in the last sum (by the same theorem applied for ${{\mathcal {T}}}_i$). Therefore, the identity (9) guarantees that for all $a_v$ large $Q^{{\mathcal {T}}}_{[l'_0],{{\mathcal {I}}}}(l'_0)$ is a quasipolynomial as well.

#### Corollary 2

For $h\in H$ fixed and $l'_0=r_h+l$, $l\in L$, if all $a_v$ are sufficiently large, $Q^{{\mathcal {T}}}_{h,{{\mathcal {I}}}}(l'_0)$ equals a quasipolynomial $\mathfrak {Q}^{{\mathcal {T}}}_{h,{{\mathcal {I}}}}(l)$ defined on *L*, where

10 $$\begin{aligned} \begin{aligned}&- \mathfrak {Q}^{{\mathcal {T}}}_{h,{{\mathcal {I}}}}(l):= \mathfrak {sw}_{-h*\sigma _{can}}(M)+\frac{(K+2r_h+2l)^2+|\mathcal {V}|}{8}\\&\quad - \sum _{i} \Big ( \mathfrak {sw}_{-[j^*_i(r_h+l)]*\sigma _{can,i}}(M_i)+\frac{(K(\mathcal {T}_i) + 2j^*_i(r_h+l))^2+ |\mathcal {V}({{\mathcal {T}}}_i)|}{8}\Big ). \end{aligned} \end{aligned}$$

Except for the ‘constant term’, $\mathfrak {Q}^{{\mathcal {T}}}_{h,{{\mathcal {I}}}}(l)$ is a multivariable quadratic polynomial. However, the constant term is a ‘periodic’ function $L\rightarrow \mathbb {Q}$. Indeed, $L\mapsto \times _i \,\mathrm{Spin}^c(M_i)$, $l\mapsto \{[j^*_i(r_h+l)]*\sigma _{can,i}\}_i$, in general, is not constant. However, if we define $\widetilde{L}\subset L$ as the kernel of the composition

11 $$\begin{aligned} L\longrightarrow \oplus _i\, L'({{\mathcal {T}}}_i) \longrightarrow \oplus _i\, L'({{\mathcal {T}}}_i)/L({{\mathcal {T}}}_i), \ \ \ l\mapsto \oplus _i [j^*_i(l)], \end{aligned}$$

then the ‘constant term’ of $\mathfrak {Q}^{{\mathcal {T}}}_{h,{{\mathcal {I}}}}(l)$ restricted to any class of type $l_0+\widetilde{L}$ of *L* is constant.

#### Corollary 3

For $h\in H$ fixed the counting function $Q^{{\mathcal {T}}}_{h,{{\mathcal {I}}}}(l'_0)$ ($l'_0\in L'_h$) of $Z_h({{\mathbf {t}}}_{{\mathcal {I}}})$ admits a quasipolynomial in the Lipman cone ${{\mathcal {S}}}'_{\mathbb {R}}$, namely $\mathfrak {Q}^{{\mathcal {T}}}_{h,{{\mathcal {I}}}}(l)$, and its periodic constant satisfies

$$\begin{aligned} \begin{aligned} -\mathrm{pc}^{{{\mathcal {S}}}'_{\mathbb {R}}}(Z_h({{\mathbf {t}}}_{{{\mathcal {I}}}}))&= - \mathfrak {Q}^{{\mathcal {T}}}_{h,{{\mathcal {I}}}}(0)= \mathfrak {sw}_{-h*\sigma _{can}}(M)+\frac{(K+2r_h)^2+|\mathcal {V}|}{8}\\&\quad - \sum _{i} \Big ( \mathfrak {sw}_{-[j^*_i(r_h)]*\sigma _{can,i}}(M_i)+\frac{(K(\mathcal {T}_i) + 2j^*_i(r_h))^2+ |\mathcal {V}({{\mathcal {T}}}_i)|}{8}\Big ). \end{aligned} \end{aligned}$$

Note that this is not the statement of Theorem 5 yet. In order to conclude Theorem 5 we make the following discussion. Above we considered $Q^{{\mathcal {T}}}_{[l'_0],{{\mathcal {I}}}}(l'_0)$, and its quasipolynomial, as functions in $l'_0\in L'_h$, or in $l=l'_0-r_h\in L$. This is the right point of view: when we take periodic constants of a sum of different quasipolynomials, one has to consider this operation in the same lattice. In this way the periodic constant will behave as an additive operator, cf. Remark  1.

However, note that $Q^{{\mathcal {T}}}_{[l'_0],{{\mathcal {I}}}}\,(l'_0)$ basically counts the coefficients of the reduced series $Z_h({{\mathbf {t}}}_{{\mathcal {I}}})$, hence it can be considered also as a counting function defined on the lattice $L|_{{\mathcal {I}}}$, via $l'_0|_{{{\mathcal {I}}}}=l|_{{\mathcal {I}}}+r_h|_{{{\mathcal {I}}}}$. In this way, its periodic constant in this lattice should be computed by substituting into $l'_0|_{{\mathcal {I}}}$ its representative in the semi-open cube associated with variables ${{\mathcal {I}}}$. But, the point is that this is exactly $r_h|_{{{\mathcal {I}}}}$ (since all the entries of $r_h|_{{\mathcal {I}}}$ are automatically in [0, 1)). Hence, the two periodic constant (computed in *L* or $L|_{{\mathcal {I}}}$) agree and provide

12 $$\begin{aligned} \mathrm{pc}^{ {{\mathcal {S}}}'_\mathbb {R}}(Z_h({{\mathbf {t}}}_{{\mathcal {I}}}))= \mathrm{pc}^{\pi _{{\mathcal {I}}}( {{\mathcal {S}}}'_\mathbb {R})}(Z_h({{\mathbf {t}}}_{{\mathcal {I}}})). \end{aligned}$$

This fact, together with Corollary 3 prove Theorem 5.

#### Remark 1

1. The analogue of (12) for the subgraphs ${{\mathcal {T}}}_i$ (that is, for the operator $j_i^*$ instead of $\pi _{{\mathcal {I}}}$) is not valid. Let us assume e.g. that $l'_0\in r_h+\widetilde{L}$, hence $[j^*_i(l'_0)]= [j^*_i(r_h)]$ is constant, say $h_i\in H({{\mathcal {T}}}_i)$. Consider the following expression valid for any $\tilde{l}'_{0}\in r_{h_i}+L({{\mathcal {T}}}_i)$ with large coefficients:
  13 $$\begin{aligned} Q^{{{\mathcal {T}}}_i}_{[\tilde{l}'_{0}]}((\tilde{l}'_{0})= -\big ((K({{\mathcal {T}}}_i)+2 \tilde{l}'_{0})^2+|{{\mathcal {V}}}({{\mathcal {T}}}_i)|\big )/8- \mathfrak {sw}_{-h_i*\sigma _{can,i}}(M_i). \end{aligned}$$
  It can be considered in two different lattices. First, the right hand side is the quasipolynomial $\mathfrak {Q}^{{{\mathcal {T}}}_i}_{h_i}(\tilde{l}'_{0}-r_{h_i})$ associated with the lattice $L({{\mathcal {T}}}_i)$. On the other hand, if we substitute into $\tilde{l}'_{0}\in L'({{\mathcal {T}}}_i)$ the restriction $j^*_i(l'_0)$, it appears as a quasipolynomial in variable $l'_0-r_h\in L({{\mathcal {T}}})$ (this expression appears in (9)). In the first case, in $L({{\mathcal {T}}}_i)$, its periodic constant is $\mathfrak {Q}^{{{\mathcal {T}}}_i}_{h_i}(r_{h_i})$, while in the second case, in the lattice *L*, it is $\mathfrak {Q}^{{{\mathcal {T}}}_i}_{h_i}(j^*_i(r_h))$. Note that usually $r_{h_i}\not =j_i^*(r_h)$, cf. Example 2. The message is the following: when we take periodic constants of a sum of different quasipolynomials, one has to consider this operation in the same lattice. However, if one of the periodic constants is needed to be reinterpreted as a periodic constant in a different lattice then one has to be aware of the fact that the $\mathrm{pc}$–operation commutes with projections of type $\pi _{{\mathcal {I}}}$, but usually not with operators of type $j_i^*$.
2. One has the following identity (for ${{\mathcal {T}}}$, and similar expressions for any ${{\mathcal {T}}}_i$)
  $$\begin{aligned}\frac{(K+2l')^2+|\mathcal {V}|}{8}= \frac{K^2+|\mathcal {V}|}{8}-\chi (l'), \end{aligned}$$
  where $\chi (l'):=-(l',l'+K)/2$ is the ‘Riemann-Roch expression’ for any $l'\in L'$.
3. For certain surgery formulae regarding the invariant $K^2+|{{\mathcal {V}}}|$ see e.g. [5, §5].

### Discussions regarding $spin^c$-structures

By different choices of $h\in H$ and of liftings $l'_0=r_h+l\in L'$, ${\widetilde{\sigma }}=l_0'*{\widetilde{\sigma }}_{can}$ gives all the possible $spin^c$–structures of $\mathrm{Spin}^c(\widetilde{X})$. ${\widetilde{\sigma }}$ extends $h*\sigma _{can}$, and its restriction to $\mathrm{Spin}^c(M_i)$ are $[j^*_i(l'_0)]*\sigma _{can,i}$. Hence the quasipolynomial identity (10), for any fixed ${\widetilde{\sigma }}$, can be regarded as a surgery formula of the Seiberg-Witten invariants connecting $(M, h*\sigma _{can})$ and $\{(M_i,[j^*_i(l'_0)] *\sigma _{can,i})\}_i$ with correction term $-\mathfrak {Q}^{{\mathcal {T}}}_{h,{{\mathcal {I}}}}(l)$, computable from the quasipolynomial of $Z^{{\mathcal {T}}}_h({{\mathbf {t}}}_{{\mathcal {I}}})$.

### Shifted series

The expression $\mathfrak {Q}^{{\mathcal {T}}}_{h,{{\mathcal {I}}}}(l)$ in Corollary 2 can be rewritten in terms of certain periodic constant computable from ${{\mathbf {t}}}_{{\mathcal {I}}}^{-l|_{{{\mathcal {I}}}}}\cdot Z_h^{{\mathcal {T}}}({{\mathbf {t}}}_{{\mathcal {I}}})$ as follows.

Assume that $S({{\mathbf {t}}})=\sum _{l\in {{\mathcal {K}}}}c^S(l){{\mathbf {t}}}^l$ is a series in variables $l\in L=\mathbb {Z}\langle E_v\rangle _v$ supported on the cone ${{\mathcal {K}}}\subset L\otimes \mathbb {R}$. We assume that $\mathcal {K}=\mathbb {R}_{\ge 0}\langle V_j\rangle _j$, where all the entries of each $V_j$ are positive. Let $Q^S(l)=\sum _{\tilde{l}\not \ge l}c^S(\tilde{l})$ be its counting function, and assume that it admits the quasipolynomial $\mathfrak {Q}^S(l)$, which satisfies $\mathfrak {Q}^S(l)=Q^S(l)$ in a shifted cone of type $l_*+{\mathcal K}$. Then, in a convenient shifted cone, for any fixed $l_0\in L$ one has

$$\begin{aligned} \mathfrak {Q}^S(l+l_0)=\sum _{\tilde{l}\not \ge l+l_0}c^S(\tilde{l})= \sum _{\tilde{l}\not \ge l}c^{{{\mathbf {t}}}^{-l_0}S}(\tilde{l}). \end{aligned}$$

Usually, ${{\mathbf {t}}}^{-l_0}S({{\mathbf {t}}})$ is not a series (it is a Laurent series), let ${{\mathbf {t}}}^{-l_0}S({{\mathbf {t}}})|_{\ge 0}$ and ${{\mathbf {t}}}^{-l_0}S({{\mathbf {t}}})|_{\not \ge 0}$ be its decomposition according to its support. Then ${{\mathbf {t}}}^{-l_0}S({{\mathbf {t}}})|_{\ge 0}$ is a series, while ${{\mathbf {t}}}^{-l_0}S({{\mathbf {t}}})|_{\not \ge 0}$ is a finite Laurent polynomial. (E.g., if $l_0\le 0$ then ${{\mathbf {t}}}^{-l_0}S|_{\not \ge 0}$ is identically zero, however, in general it is not.) Furthermore, for *l* with large coefficients, $\sum _{\tilde{l}\not \ge l}c^{{{\mathbf {t}}}^{-l_0}S|_{\not \ge 0}}(\tilde{l})=({{\mathbf {t}}}^{-l_0}S|_{\not \ge 0})(\mathbf{1})$ (i.e. one substitutes for each $t_v=1$). This proves the following fact.

#### Proposition 1

Under the above notations, for any $l_0\in L$ the series ${{\mathbf {t}}}^{-l_0}S({{\mathbf {t}}})|_{\ge 0}$ admits a quasipolinomial and a periodic constant in the cone $\mathcal {K}$ and

$$\begin{aligned} \mathfrak {Q}^S(l_0)=({{\mathbf {t}}}^{-l_0}S|_{\not \ge 0})(\mathbf{1})+\mathrm{pc}^{\mathcal {K}} ({{\mathbf {t}}}^{-l_0}S|_{\ge 0}). \end{aligned}$$

Using this identity, Corollary 2 (and 3.4 as well) can be modified accordingly.

### Modified counting functions

We say that $a,b\in \mathbb {R}^k$ satisfies $a\prec b$ if for *all* coordinates we have $a_v<b_v$. By inclusion–exclusion principle, a sum of type $Q^{{\mathcal {T}}}_{[l'_0],{{\mathcal {I}}}}\,(l'_0)$ can be rewritten as

$$\begin{aligned} \sum _{l'|_{{{\mathcal {I}}}}\not \ge l'_0|_{{{\mathcal {I}}}}} z^{{\mathcal {T}}}(l') =\sum _{\exists w\in {{\mathcal {I}}}\,:\, l'|_{w}< (l'_{0})|_w} z^{{\mathcal {T}}}(l')= \sum _{\emptyset \not ={{\mathcal {J}}}\subset {{\mathcal {I}}}} \ (-1)^{|{{\mathcal {J}}}|+1} \sum _{l'|_{{\mathcal {J}}}\prec l'_0|_{{\mathcal {J}}}} \ z^{{\mathcal {T}}}(l'), \end{aligned}$$

where everywhere in the summations $[l']=[l_0']$. This motivates to define (for $l'_0\in L'$ with $[l'_0]=h$) the ‘modified counting functions’

$$\begin{aligned} q^{{\mathcal {T}}}_{h,{{\mathcal {J}}}} \, (l'_0):=\sum _{l'|_{{\mathcal {J}}}\prec \, l'_{0}|_{{\mathcal {J}}}, \ [l']=[l'_0]} \ z^{{\mathcal {T}}}(l'). \end{aligned}$$

There are similar expressions for the terms $Q^{{{\mathcal {T}}}_i}_{[j^*_i(l'_0)]}(j^*_i(l'_0))$ of (9) as well. Hence, we can rewrite the desired identities in terms of modified counting functions. The point is that we will prove the corresponding identities for these modified counting functions. Their advantage is that they satisfy certain ‘convexity’ properties, which generate a lot of cancellations. They will be treated in Sect. 4, a part which constitutes also the start of the proof of (9).

#### Remark 2

In [16] the expression $q^{{\mathcal {T}}}_{h,{{\mathcal {I}}}} \, (l'_0)$ is called the ‘coefficient function’, since it is the coefficient of ${\mathbf {t}}^{l'_0}_{{{\mathcal {I}}}}$ in the Taylor expansion of $f_h({\mathbf {t}}_{{{\mathcal {I}}}})\cdot \prod _{v\in {{\mathcal {I}}}}{\mathbf {t}}^{E_v}_{{{\mathcal {I}}}}/(1-{\mathbf {t}}^{E_v}_{{{\mathcal {I}}}})$.

## A ‘convexity’ property of the modified counting functions

### Some terminology. Multiplicity systems.

Assume that ${{\mathcal {T}}}$ is a graph as above, and $l=\sum _vm_vE_v\in L$ is an integral cycle, which in the dual base is $l=\sum _vc_vE^*_v$. For each $v\in {{\mathcal {V}}}$ in $\widetilde{X}$ we consider 2–discs (cuts) $\{C_{v,i}\}_{i=1}^{k_v}$, each of them intersecting $E_v$ transversally in generic points and with $\partial C_{v,i}\subset \partial \widetilde{X}$. Then, whenever $\sum _i c_{v,i}=c_v$ for all *v*, $C(l) :=\sum _v (m_vE_v+\sum _ic_{v,i}C_{v,i})$ is a relative cycle in $\widetilde{X}$ with $(C(l),E_v)=0$ for all *v* (hence its class in $H_2(\widetilde{X}, \partial \widetilde{X},\mathbb {Z})$ is zero). Each $\partial C_{v,i}\subset \partial \widetilde{X}$ is a link component in $\partial \widetilde{X}$, and their collection endowed with the multiplicities $\{c_{v,i}\}_{v,i}$ forms a multilink with ‘*multiplicity system*’ $\{m_v,c_{v,i}\}_{v,i}$ [9], see also [36] for the non–integral homology sphere case.

If $c_{v,i}\ge 0$ and at least one inequality is strict, then each $m_v>0$ too (use the fact that the entries of $E^*_v$ are positive). Moreover, under the same hypothesis, the multilink is fibered.

Fix *C*(*l*) and a multiplicity system as above. Let ${{\mathcal {T}}}'$ be a full connected subgraph of ${{\mathcal {T}}}$ and $\widetilde{X}({{\mathcal {T}}}')$ a small tubular neighbourhood of $\cup _{v\in {{\mathcal {V}}}({{\mathcal {T}}}')}E_v$ in $\widetilde{X}$. Then *C*(*l*) induces a homologically trivial relative cycle $C(l)\cap \widetilde{X}({{\mathcal {T}}}')$ (by multiplicity preserving intersection) in $\widetilde{X}({{\mathcal {T}}}')$, hence a multiplicity system of ${{\mathcal {T}}}'$. In a different language, the $E_v$, respectively the $E_v^*$–multiplicities of *l* after restriction are the following. A cut $C_v$ is preserved with its multiplicity $c_v$ if $v\in {{\mathcal {V}}}({{\mathcal {T}}}')$, otherwise it becomes empty. The restriction of $E_v$ becomes empty if $E_v\cap E({{\mathcal {T}}}')=\emptyset$, it becomes a cut of $E_w$ with multiplicity $m_v$ if $E_v\cap E({{\mathcal {T}}}')$ is the point $E_v\cap E_w$, and it remains $E_v$ with its multiplicity $m_v$ if $v\in {{\mathcal {V}}}({{\mathcal {T}}}')$. (Homologically, this is the operator $j^*$ associated with the inclusion $j:L({{\mathcal {T}}}')\hookrightarrow L({{\mathcal {T}}})$.)

### Splitting graphs

Fix ${{\mathcal {T}}}$ as in  2.1. Let ${{\mathcal {T}}}_2$ be a connected full subgraph of ${{\mathcal {T}}}$ with vertices ${{\mathcal {V}}}_2$ and we define its ‘boundary’ as

$$\begin{aligned} {{\mathcal {B}}}:=\{u\in {{\mathcal {V}}}_2\,:\ \exists \ w\not \in {{\mathcal {V}}}_2 \ \text{ adjacent } \text{ to } \text{ u } \text{ in } {{\mathcal {T}}}\}. \end{aligned}$$

For any fixed $u\in {{\mathcal {B}}}$, ${{\mathcal {T}}}_{1,u}$ denotes that full connected subgraph of ${{\mathcal {T}}}$, which contains *u* and all the connected components of ${{\mathcal {T}}}{\setminus }{{\mathcal {T}}}_2$, which have adjacent vertices with *u*. Write ${{\mathcal {V}}}_{1,u}={{\mathcal {V}}}({{\mathcal {T}}}_{1,u})$.

As above, $Z({{\mathbf {t}}}_{{{\mathcal {V}}}_2})$ is the reduction of the series of ${{\mathcal {T}}}$ to the variables indexed by ${{\mathcal {V}}}_2$.

For simplicity, we use the same notation $l'|_u$ for the $E_u$-coefficient of $l'\in L'$ too (cf. 2.5).

#### Lemma 1

- Any element from the support of $Z({{\mathbf {t}}}_{{{\mathcal {V}}}_2})$ can be written in a unique way as $\sum _{v\in {{\mathcal {V}}}_2} r_vE^*_v|_{{{\mathcal {V}}}_2}$ for certain coefficients $r_v\in \mathbb {Q}_{\ge 0}$.
- Fix $u\in {{\mathcal {B}}}$. Let $\delta _{2,u}$ be the number of edges adjacent to *u* but sitting in ${{\mathcal {T}}}_2$. Assume that $\delta _{2,u}\ge 2$. If $\sum _{v\in {{\mathcal {V}}}_2} r_vE^*_v|_{{{\mathcal {V}}}_2}$ is in the support of $Z({{\mathbf {t}}}_{{{\mathcal {V}}}_2})$, then $r_u\cdot E^*_u|_u\le \sum _{v\in {{\mathcal {V}}}_{1,u}} (\delta _v-2)E^*_v|_u$.

#### Proof

- For $u\in {{\mathcal {B}}}$ and $v\in {{\mathcal {V}}}_{1,u}$, the cycle $E^*_v\cdot E^*_u|_u- E^*_u\cdot E^*_v|_u\in L\otimes \mathbb {Q}$ is supported in ${{\mathcal {V}}}_{1,u}{\setminus }u$. (Indeed, the $E_u$–multiplicity of $l:=E^*_v\cdot E^*_u|_u- E^*_u\cdot E^*_v|_u$ is zero. Therefore, the restriction of the multiplicity system *C*(*l*) to ${{\mathcal {V}}}_{1,u}{\setminus }u$ has no cuts, hence it is identically zero, cf. 4.1.) In particular,
  14 $$\begin{aligned} E^*_v|_{{{\mathcal {V}}}_2}= E^*_u|_{{{\mathcal {V}}}_2}\cdot (E^*_v|_u/ E^*_u|_u). \end{aligned}$$
  Next, write $Z({{\mathbf {t}}})$ as $Z_2({{\mathbf {t}}})\cdot \prod _{u\in {{\mathcal {B}}}}Z_{1,u}({{\mathbf {t}}})$, where $Z_{1,u}({{\mathbf {t}}}):= \prod _{v\in {{\mathcal {V}}}_{1,u}} (1-{{\mathbf {t}}}^{E^*_v})^{\delta _v-2}$ and $Z_2({{\mathbf {t}}}):= \prod _{v\in {{\mathcal {V}}}_2{\setminus }{{\mathcal {B}}}} (1-{{\mathbf {t}}}^{E^*_v})^{\delta _v-2}$. Hence, in the support of $Z({{\mathbf {t}}}_{{{\mathcal {V}}}_2})$, $Z_2({{\mathbf {t}}}_{{{\mathcal {V}}}_2})$ contributes with $\{E_v^*|_{{{\mathcal {V}}}_2}\}_{v\in {{\mathcal {V}}}_2\setminus {{\mathcal {B}}}}$, while, $Z_{1,u}({{\mathbf {t}}}_{{{\mathcal {V}}}_2})$ with $E_u^*|_{{{\mathcal {V}}}_2}$ for each $u\in {{\mathcal {B}}}$. Moreover, $\{E^*_v|_{{{\mathcal {V}}}_2}\}_{v\in {{\mathcal {V}}}_2}$ are linearly independent. Indeed, if $\sum _{v\in {{\mathcal {V}}}_2}r_vE^*_v|_{{{\mathcal {V}}}_2}=0$ then $x:=\sum _{v\in {{\mathcal {V}}}_2}r_vE^*_v$ is supported in ${{\mathcal {V}}}\setminus {{\mathcal {V}}}_2$, but $(x,E_v)=0$ for any $v\in {{\mathcal {V}}}{\setminus }{{\mathcal {V}}}_2$; hence $x=0$ since the intersection form of any subgraph is non–degenerate, see Lemma 2 too.
- We construct the following graph ${{\mathcal {T}}}_{1,u}'$ with arrowheads: ${{\mathcal {T}}}_{1,u}'$ consists of all the vertices and edges of ${{\mathcal {T}}}_{1,u}$, and we also add $\delta _{2,u}$ arrowheads attached to *u* (that is, we replace the *u*–adjacent edges from ${{\mathcal {T}}}_2$ by arrowheads). Each arrowhead represent a cut (of $E_u$) in $\widetilde{X}({{\mathcal {T}}}_{1,u})$. We regard their collection as a multilink, that is, we endow the vertices and arrowheads with a multiplicity system (of ${{\mathcal {T}}}_{1,u}'$) as in 4.1. We define the $m_v$–multiplicities as the multiplicities of $dE_u^*$ restricted to ${{\mathcal {V}}}_{1,u}$, where $d=\det (-(\,,\,)_{{\mathcal {T}}})$. That is, the multiplicity of a vertex *v* is $dE^*_u|_v=dE^*_v|_u=-d(E^*_v,E^*_u)\in \mathbb {Z}_{>0}$. Then the sum of the multiplicities of the arrowheads (all of them at *u*) should be $c_u=d+d\sum _w E^*_u|_w$, where the sum runs over the adjacent vertices *w* of *u* in ${{\mathcal {T}}}_2$ (there are $\delta _{2,u}$ of them). Since each $dE^*_u|_w\ge 1$, we get $c_u\ge d+\delta _{2,u}$, hence we can distribute $c_u$ into $\delta _{2,u}$ positive integers, such that one of them is 1. These integers will be the multiplicities of the arrowheads (link components). The constructed multiplicity system defines a fibred multilink (cf. [9]). Since one of the multiplicities is 1, the corresponding Milnor fiber is connected (see also [9, Th. 11.3]). Furthermore, the monodromy zeta function of the Milnor fibration is (by A’Campo’s theorem [1] or [9]) $\zeta (t)=\prod _{v\in {{\mathcal {V}}}_{1,u}}(1-t^{dE^*_v|_u})^{\delta _v-2}$. Hence, comparing the definitions of $\zeta$ and $Z_{1,u}$, and using (14), we get that the reduced series of $Z_{1,u}$ is obtained by the following substitution:
  15 $$\begin{aligned} Z_{1,u}({{\mathbf {t}}}_{{{\mathcal {V}}}_2})=\zeta (t)|_{t\mapsto {{\mathbf {t}}}_{{{\mathcal {V}}}_2}^{E^*_u|_{{{\mathcal {V}}}_2}/ dE^*_u|_u}}.\end{aligned}$$
  We claim that if $\delta _{2,u}\ge 2$ then $\zeta (t)$ is a polynomial. Indeed, being a zeta function of a connected Milnor fiber, it has the form $\varDelta (t)/(t-1)$, where $\varDelta (t)$ is the characteristic polynomial of the monodromy of the first homology of the Milnor fiber. Hence, $\zeta$ is a polynomial if and only if it has no pole at $t=1$. But, since ${{\mathcal {T}}}_{1,u}$ is a tree, the vanishing order $\mathrm{ord}_{t-1}\zeta (t)=\sum _{v\in {{\mathcal {V}}}_{1,u}}(\delta _v-2)=-2+\delta _{2,u} \ge 0$. Furthermore, the degree of $\zeta$ is $\sum _{v\in {{\mathcal {V}}}_{1,u}} (\delta _v-2)dE^*_v|_u$, and the (rational) $E^*_u|_{{{\mathcal {V}}}_2}$ degree of $Z_{1,u}({{\mathbf {t}}}_{{{\mathcal {V}}}_2})$ is $\deg (\zeta )/(dE^*_u|_u)$. Finally, by (a) and its proof, all contribution in the coefficient of $E^*_u|_{{{\mathcal {V}}}_2}$ in $Z({{\mathbf {t}}}_{{{\mathcal {V}}}_2})$ comes from $Z_{1,u}({{\mathbf {t}}}_{{{\mathcal {V}}}_2})$. $\square$

### The modified intersection form

Recall that the cycles $\{-E^*_v\}_{v\in {{\mathcal {V}}}}$, considered as column vectors of a matrix, form the inverse $(\,,\,)^{-1}$ of the intersection form. A similar property is valid for the restrictions $\{-E^*_v|_{{{\mathcal {V}}}_2}\}_{v\in {{\mathcal {V}}}_2}$.

For a graph ${{\mathcal {T}}}$ we say that a bilinear form $(\,,\,)_{mod}$ of $L\otimes \mathbb {Q}$ is a *modified intersection form* of $(\,,\,)=(\,,\,)_{{\mathcal {T}}}$, if $(E_v,E_w)_{mod}=(E_v,E_w)$ for any $v\not =w$ (and the diagonal might be modified, usually into some rational entries).

#### Lemma 2

[16, Lemma 11 (iii)] Let ${{\mathcal {V}}}_2$ be as in 4.2. The $|{{\mathcal {V}}}_2|$–rank matrix $\{-E^*_v|_{{{\mathcal {V}}}_2}\}_{v\in {{\mathcal {V}}}_2}$ is the inverse of a negative definite matrix $(\,,\,)_{mod}$, a modified intersection form of $(\,,\,)_{{{\mathcal {T}}}_2}$. (In fact, all the diagonal entries, which are modified are indexed by ${{\mathcal {B}}}$.)

The proof is based on a diagonalization procedure of $(\,,\,)_{{\mathcal {T}}}$ from [9, §21].

### The convexity property

Let ${{\mathcal {T}}}$ be as in 4.2, and let us fix a subset ${{\mathcal {I}}}\subset {{\mathcal {V}}}$, ${{\mathcal {I}}}\not =\emptyset$. The closure ${\overline{{{\mathcal {I}}}}}$ of ${{\mathcal {I}}}$ is defined as the set of vertices of that connected minimal full subgraph of ${{\mathcal {T}}}$ which contains ${{\mathcal {I}}}$.

The following proposition was first proved (with slightly weaker bound) in [16] using residue formulae for vector partitions of [38]. Here we provide an independent proof.

#### Proposition 2

Assume that $l_0'\in \sum _v(\delta _v-2)E^*_v+{{\mathcal {S}}}'$, $[l'_0]=h$. Then $q^{{\mathcal {T}}}_{h,{{\mathcal {I}}}} (l'_0)=q^{{\mathcal {T}}}_{h,{\overline{{{\mathcal {I}}}}}} (l'_0)$.

#### Proof

Let us write ${{\mathcal {T}}}_2$ for the full connected subgraph with ${{\mathcal {V}}}_2={{\mathcal {V}}}({{\mathcal {T}}}_2)={\overline{{{\mathcal {I}}}}}$, and we adopt the notations of 4.2 associated with ${{\mathcal {T}}}_2$. Furthermore, we use the following notations as well: $l'_0=\sum _va_vE^*_v$ is the fixed element of $L'$ appearing in the statement, $l'=\sum _vb_vE^*_v$ is an element from the support $\mathrm{Supp}(Z_h)$ of $Z_h$ (i.e. $z^{{\mathcal {T}}}(l')\not =0$), and $l=l'-l'_0\in L$ with $l|_{{{\mathcal {I}}}}\prec 0$. (Such $l'$ parametrize the support of the sum in $q^{{\mathcal {T}}}_{h,{{\mathcal {I}}}} (l'_0)$.) Write $c_v=b_v-a_v$ and $l=\sum _vm_vE_v$ (hence $\{m_v,c_v\}_v$ is a multiplicity system in the sense of 4.1). The assumption $l|_{{{\mathcal {I}}}}\prec 0$ reads as $m_v<0$ for all $v\in {{\mathcal {I}}}$.

We wish to compare the sets $\{l'\in \mathrm{Supp}(Z_h)\,:\, (l'-l'_0)|_{{{\mathcal {I}}}} \prec 0\}$ and $\{l'\in \mathrm{Supp}(Z_h)\,:\, (l'-l'_0)|_{{\overline{{{\mathcal {I}}}}}} \prec 0\}$ for fixed $l'_0$. If they agree then definitely we get $q^{{\mathcal {T}}}_{h,{{\mathcal {I}}}} (l'_0)=q^{{\mathcal {T}}}_{h,{\overline{{{\mathcal {I}}}}}} (l'_0)$ (since we sum over the same set). The point is that these two sets can be different, however, we show that the sum of the coefficients over the support–difference is zero.

To start the proof, let us fix some $l'\in \mathrm{Supp}(Z_h)$ with $(l'-l'_0)|_{{{\mathcal {I}}}} \prec 0$.

First, we check an easy inequality. Let us take $v\in {{\mathcal {V}}}$ with $\delta _v>1$. Then, by the assumption of Proposition 2, $a_v\ge \delta _v-2$. But, by the shape of the rational function $f({{\mathbf {t}}})$ from (1), $b_v\le \delta _v-2$. Hence

16 $$\begin{aligned} c_v\le 0 \ \ \text{ whenever } \ \ \delta _v>1. \end{aligned}$$

*The proof in the ‘easy case’.* Assume that $m_u<0$ for all $u\in {{\mathcal {B}}}$. We claim that $m_v<0$ for all $v\in \overline{{{\mathcal {I}}}}$, hence $q^{{\mathcal {T}}}_{h,{{\mathcal {I}}}} (l'_0)=q^{{\mathcal {T}}}_{h,{\overline{{{\mathcal {I}}}}}} (l'_0)$ by the above discussion.

Assume that this is not the case, and choose a maximal connected full subgraph ${{\mathcal {T}}}'$ of ${{\mathcal {T}}}_2$ with all $m_v$–multiplicities non–negative.

Let $C(l)=\sum _v(m_vE_v+c_vC_v)$ be the homologically trivial relative cycle in $\widetilde{X}$ associated with *l* (with some choices of cuts $C_v$) as in 4.1. Then *C*(*l*) induces a relative cycle and a multiplicity system of ${{\mathcal {T}}}'$ via the multiplicity preserving intersection $C(l)\cap \widetilde{X}({{\mathcal {T}}}')$, as it is explained in 4.1.

By construction ${{\mathcal {V}}}({{\mathcal {T}}}')\subset {\overline{{{\mathcal {I}}}}}\setminus {{\mathcal {I}}}$ and for all $v\in {{\mathcal {V}}}({{\mathcal {T}}}')$ one has $\delta _v>1$. Therefore, those cut–multiplicities which come as restrictions of cuts of *C*(*l*) are $\le 0$ by (16). The other cut–multiplicities, which come from the restriction of some neighboring $E_v$’s have multiplicities $m_v<0$ (by the maximality of ${{\mathcal {T}}}'$). Therefore, the restriction of *C*(*l*) to ${{\mathcal {T}}}'$ has all cut–multiplicities $\le 0$, with at least one $<0$ (at the ‘boundary’ of ${{\mathcal {T}}}'$). On the other hand, all $E_v$–multiplicities $\ge 0$. These facts contradict the last sentence of 4.1. $\square$

*The proof in the general case.* Assume that $m_u\ge 0$ for at least one $u\in {{\mathcal {B}}}$.

Let us define the rational coefficients $\{c_{1,u}\}_{u\in {{\mathcal {V}}}_2}$ as follows:

17 $$\begin{aligned} c_{1,u}:=\left\{ \begin{array}{cc} \sum _{v\in {{\mathcal {V}}}_{1,u}} c_v E^*_v|_u/ E^*_u|_u &{} \ \text{ if } \ u\in {{\mathcal {B}}}\\ c_u &{} \ \,\text{ if } \ u\not \in {{\mathcal {B}}}.\end{array}\right. \end{aligned}$$

Then, by (14), for each $u\in {{\mathcal {B}}}$, $\sum _{v\in {{\mathcal {V}}}_{1,u}} c_v E^*_v|_{{{\mathcal {V}}}_2}= c_{1,u} E^*_u|_{{{\mathcal {V}}}_2}$, hence

18 $$\begin{aligned} \sum _{v\in {{\mathcal {V}}}}c_vE^*_v|_{{{\mathcal {V}}}_2}=\sum _{v\in {{\mathcal {V}}}_2}c_{1,v}E^*_v|_{{{\mathcal {V}}}_2}= \sum _{v\in {{\mathcal {V}}}_2}m_vE_v|_{{{\mathcal {V}}}_2}=l|_{{{\mathcal {V}}}_2}. \end{aligned}$$

#### Claim

There exists $u\in {{\mathcal {B}}}$ with $m_u\ge 0$, $c_{1,u}>0$ and $\delta _{2,u}\ge 2$. (For the definition of $\delta _{2,u}$ see Lemma 1(b).)

#### Proof

Set ${{\mathcal {V}}}_2^{<0}:=\{v\in {{\mathcal {V}}}_2\,:\, m_v< 0\}$ and ${{\mathcal {V}}}_2^{\ge 0}:=\{v\in {{\mathcal {V}}}_2\,:\, m_v\ge 0\}$. By assumptions ${{\mathcal {I}}}\subset {{\mathcal {V}}}_2^{<0}$ and ${{\mathcal {V}}}_2^{\ge 0}\not =\emptyset$ too. Write $l|_{{{\mathcal {V}}}_2}$ as $l_1-l_2$, $l_1$ supported in ${{\mathcal {V}}}_2^{\ge 0}$, while $l_2$ supported in ${{\mathcal {V}}}_2^{< 0}$, both effective. Consider also the negative definite modified intersection form $(\,,\,)_{mod}$ associated with $\{-E^*_v|_{{{\mathcal {V}}}_2}\}_{v\in {{\mathcal {V}}}_2}$, defined in Lemma 2. If $l_1\not =0$ then $(l|_{{{\mathcal {V}}}_2},l_1)_{mod}\le (l_1,l_1)_{mod}<0$, hence there exists $u\in {{\mathcal {V}}}_2^{\ge 0}$ (in the support of $l_1$) such that $(l|_{{{\mathcal {V}}}_2},E_u)_{mod}<0$. If $l_1=0$, since ${{\mathcal {T}}}_2$ is connected, one can find $u\in {{\mathcal {V}}}_2^{\ge 0}$ such that $E_u$ intersects the support of $l|_{{{\mathcal {V}}}_2}$, hence $(l|_{{{\mathcal {V}}}_2},E_u)_{mod}<0$ again. But, via (18), $(l|_{{{\mathcal {V}}}_2},E_u)_{mod}=-c_{1,u}$. Hence, there exists $u\in {{\mathcal {V}}}_2^{\ge 0}$ such that $c_{1,u}>0$. Using (16) and definition (17) we get that $u\in {{\mathcal {B}}}$ necessarily. On the other hand, $\delta _{2,u}\ge 2$ too. Indeed, if $u\in {{\mathcal {B}}}$ and $\delta _{2,u}=1$ then $u\in {{\mathcal {I}}}$ (since ${{\mathcal {V}}}_2={\overline{{{\mathcal {I}}}}}$ is the closure of ${{\mathcal {I}}}$) hence $m_u<0$. $\square$

Let us introduce the coefficients $\{b_{1,v}\}_{v\in {{\mathcal {V}}}_2}$ associated with $\{b_v\}_{v\in {{\mathcal {V}}}}$ by similar definitions as (17). The assumption regarding $a_v$’s, and $c_{1,u}>0$, we obtain that the $E^*_u|_{{{\mathcal {V}}}_2}$–coefficient $b_{1,u}$ of $l'|_{{{\mathcal {V}}}_2}$ satisfies

$$\begin{aligned} b_{1,u}\!=\! \sum _{v\in {{\mathcal {V}}}_{1,u}} b_v E^*_v|_u/ E^*_u|_u > \!\sum _{v\in {{\mathcal {V}}}_{1,u}} a_v E^*_v|_u/ E^*_u|_u\ge d_{1,u}:=\! \sum _{v\in {{\mathcal {V}}}_{1,u}} (\delta _v-2) E^*_v|_u/ E^*_u|_u. \end{aligned}$$

In particular, by Lemma 1, $l'|_{{{\mathcal {V}}}_2}$ is not in the support of $Z_h({{\mathbf {t}}}_{{{\mathcal {V}}}_2})$.

This fact can be reorganized as follows. We order $\{u\in {{\mathcal {B}}}\,:\, \delta _{2,u}\ge 2\} =\{u_1,\ldots ,u_s\}$. We set

$$\begin{aligned} \mathrm{Supp}_1:= \{l'\,:\, [l']=[l'_0],\ l'|_{{{\mathcal {I}}}}\prec l'_0|_{{{\mathcal {I}}}}, \ b_{1,u_1}>d_{1,u_1}\} \end{aligned}$$

and for $$s\ge j>1$$

$$\begin{aligned} \mathrm{Supp}_j:= \{l'\,:\, [l']=[l'_0],\ l'|_{{{\mathcal {I}}}}\prec l'_0|_{{{\mathcal {I}}}}, \ b_{1,u_k}\le d_{1,u_k} \ \text{ for } k<j ,\ \ b_{1,u_j}>d_{1,u_j}\}. \end{aligned}$$

Consider the restriction function $\pi _j:\mathrm{Supp}_j\rightarrow L({{\mathcal {T}}}_2)\otimes \mathbb {Q}$, $l'\mapsto l'|_{{{\mathcal {V}}}_2}$. Then the sum $\sum z^{{\mathcal {T}}}(l')$ over any of the fiber of $\pi _j$ is zero. Indeed, if we write $Z({{\mathbf {t}}}_{{{\mathcal {V}}}_2})$ as $Z_2({{\mathbf {t}}}_{{{\mathcal {V}}}_2})\cdot \prod _{u\in {{\mathcal {B}}}} Z_{1,u}({{\mathbf {t}}}_{{{\mathcal {V}}}_2})$, as in the proof of Lemma 1, then $Z_{1,u_j}({{\mathbf {t}}}_{{{\mathcal {V}}}_2})$ collects the contribution from ${{\mathcal {T}}}_{1,u_j}$ (as in the proof of 1). Then in the fiber of $\pi _j$ the coefficient $b_{1,u_j}$ is larger than the $E^*_{u_j}|_{{{\mathcal {V}}}_2}$–degree of $Z_{1,u_j}({{\mathbf {t}}}_{{{\mathcal {V}}}_2})$, and by Lemma 1(b) $l'|_{{{\mathcal {V}}}_2}$ is not in the support of $Z_h({{\mathbf {t}}}_{{{\mathcal {V}}}_2})$. This means that the sum of the corresponding coefficients is zero. In particular, the corresponding sum over all $\mathrm{Supp}_j$ is zero for any *j*.

Hence, up to these zero sums in the ‘modified counting function’, we can consider only the cycles $l'$ from $\{ \mathrm{Supp}(Z_h)\,:\, (l'-l'_0)|_{{{\mathcal {I}}}} \prec 0\}{\setminus }\cup _j \mathrm{Supp}_j$. But by the above discussion such a cycle satisfies $m_u<0$ for all $u\in {{\mathcal {B}}}$, hence $m_u<0$ for all $u\in {\overline{{{\mathcal {I}}}}}$ (by the ‘easy case’). Hence $q^{{\mathcal {T}}}_{h,{{\mathcal {I}}}} (l'_0)=q^{{\mathcal {T}}}_{h,{\overline{{{\mathcal {I}}}}}} (l'_0)$.

## A surgery formula for modified counting functions

### The main technical lemma

Choose some $v\in {{\mathcal {V}}}$, and let ${{\mathcal {T}}}{\setminus }v=\cup _{k} {{\mathcal {T}}}_{v,k}$ be the connected components of ${{\mathcal {T}}}{\setminus }v$. Let $j^*_{v,k}:L'({{\mathcal {T}}})\rightarrow L'({{\mathcal {T}}}_{v,k})$ be the dual operator defined similarly as $j^*_i$ above.

#### Lemma 3

Fix one of the components, say ${{\mathcal {T}}}_{v,k'}$, and let ${{\mathcal {J}}}\subset {{\mathcal {V}}}({{\mathcal {T}}}_{v,k'})$, ${{\mathcal {J}}}\not =\emptyset$. Then for any $l_0'\in \sum _v(\delta _v-2)E^*_v+{{\mathcal {S}}}'$ one has

19 $$\begin{aligned} q^{{\mathcal {T}}}_{h,{{\mathcal {J}}}}\,(l'_0)-q^{{\mathcal {T}}}_{h,{{\mathcal {J}}}\cup v}\,(l'_0)= q^{{{\mathcal {T}}}_{v,k'}}_{[j^*_{v,k'}(l'_0)],{{\mathcal {J}}}}\,(j^*_{v,k'}(l'_0)). \end{aligned}$$

#### Proof

We will prove Lemma 3 in three steps.

#### Step 1

First we assume that *v* is an end–vertex of ${{\mathcal {T}}}$, and the adjacent vertex *w* has $\delta _w=2$. Partly, we will follow the strategy of the proof of Proposition 3.2.4 of [25]. We will write $j^*$ for the dual operator, and we will use the notations of the proof of Proposition  2: $l'=\sum _ub_uE^*_u$, $l'_0=\sum _ua_uE^*_u$, $l=l'-l'_0\in L$, $l=\sum _uc_uE^*_u=\sum _um_uE_u$. Define also $Supp({{\mathcal {T}}})= \{l'\,:\, l|_{{{\mathcal {J}}}}\prec 0,\ l|_v\ge 0\}\cap Supp(Z^{{\mathcal {T}}}({{\mathbf {t}}}))$, $Supp({{\mathcal {T}}}{\setminus }v)= \{\tilde{l}'\,:\, \tilde{l}'|_{{{\mathcal {J}}}}\prec j^*(l'_0)|_{{{\mathcal {J}}}},\ [\tilde{l}']=[ j^*(l'_0)]\}\cap Supp(Z^{{{\mathcal {T}}}{\setminus }v} ({{\mathbf {t}}}_{{{\mathcal {V}}}{\setminus }v}))$. In the left (resp. right) hand side of (19) we sum over $Supp({{\mathcal {T}}})$ (resp. $Supp({{\mathcal {T}}}{\setminus }v)$).

In order to identify the coefficients of $Z^{{\mathcal {T}}}({{\mathbf {t}}})$ and $Z^{{{\mathcal {T}}}{\setminus }v}({{\mathbf {t}}}_{{{\mathcal {V}}}{\setminus }v})$ easier it is convenient to make the change of variables $x_u:={{\mathbf {t}}}^{E^*_u}$ ($u\in {{\mathcal {V}}}$), hence $Z^{{\mathcal {T}}}({{\mathbf {t}}})$ becomes $Z^{{\mathcal {T}}}({{\mathbf {x}}})=\prod _u(1-x_u )^{\delta _u-2}$. In particular,

20 $$\begin{aligned} Z^{{\mathcal {T}}}({{\mathbf {x}}})=Z_0({{\mathbf {x}}}_0)\cdot (1-x_v)^{-1}, \ \ \text{ and } \ \ \ Z^{{{\mathcal {T}}}{\setminus }v}({{\mathbf {x}}})=Z_0({{\mathbf {x}}}_0)\cdot (1-x_w)^{-1}, \end{aligned}$$

where $Z_0({{\mathbf {x}}}_0)$ is a series in variable $\{x_u\}_{u\in {{\mathcal {V}}}{\setminus }\{v,w\}}$ only.

Since $\delta _w=2$, if $l'\in Supp(Z^{{\mathcal {T}}}({{\mathbf {t}}}))$ then $b_w=0$.

If we apply $j^*$ to the identity $l'-l'_0=l$ we get

$$\begin{aligned} j^*(l')-j^*(l'_0)=-m_vE^*_w+\sum _{u\in {{\mathcal {V}}}{\setminus }v}m_uE_u. \end{aligned}$$

Hence, $\varPhi :Supp({{\mathcal {T}}})\rightarrow Supp({{\mathcal {T}}}{\setminus }v)$, $l'\mapsto j^*(l')+m_vE^*_w$ is well–defined. Write ${\bar{l}}'=\sum _{u\not =w,v}b_uE^*_u$, ${\bar{l}}'_0=\sum _{u\not =w,v}a_uE^*_u$. Then $l'={\bar{l}}'+b_vE^*_v$ and $\varPhi (l')={\bar{l}}'+m_vE^*_w$. Since $m_v=-(l,E^*_v)$ one has

21 $$\begin{aligned} m_v=-({\bar{l}}'-{\bar{l}}'_0,E^*_v)+a_w(E^*_w,E^*_v)-(b_v-a_v)(E^*_v,E^*_v). \end{aligned}$$

Hence $$\varPhi $$ (that is, $$({\bar{l}}',b_v)\mapsto ({\bar{l}}',m_v)$$) is injective. Furthermore, for any $$({\bar{l}}',m_v)\in Supp({{\mathcal {T}}}{\setminus }v)$$ the equation (21) provides a unique well–defined candidate for $$b_v$$, such that $$({\bar{l}}',b_v)$$ satisfies all the requirement of the elements of $$Supp({{\mathcal {T}}})$$ except maybe $$b_v\ge 0$$ (cf. (20)). Define $$Supp({{\mathcal {T}}}{\setminus }v)^{\ge 0}$$ as a subset of $$Supp({{\mathcal {T}}}{\setminus }v)$$ consisting of those elements $$({\bar{l}}',m_v)$$ for which $$b_v $$ computed via (21) is $$\ge 0$$; that is, $$Supp({{\mathcal {T}}}{\setminus }v)^{\ge 0}=\mathrm{im}(\varPhi )$$. Then, using the bijection $$\varPhi $$ onto its image and (20)

$$\begin{aligned} \sum _{l'\in Supp({{\mathcal {T}}})}\ z^{{\mathcal {T}}}(l')\ =\ \sum _{\tilde{l}'\in Supp({{\mathcal {T}}}{\setminus }v)^{\ge 0}} \ z^{{{\mathcal {T}}}{\setminus }v} (\tilde{l}'). \end{aligned}$$

Set $Supp({{\mathcal {T}}}{\setminus }v)^{< 0}:=Supp({{\mathcal {T}}}{\setminus }v)\setminus Supp({{\mathcal {T}}}{\setminus }v)^{\ge 0}$. In order to finish the proof of (19), we need

22 $$\begin{aligned} \sum _{\tilde{l}'\in Supp({{\mathcal {T}}}{\setminus }v)^{<0}} \ z^{{{\mathcal {T}}}{\setminus }v} (\tilde{l}')=0. \end{aligned}$$

This follows similarly as the vanishings from the second part of the proof of Proposition 2.

Using Proposition 2 and its proof, we can assume that ${{\mathcal {J}}}=\overline{{{\mathcal {J}}}}$. Let $u_0$ be that vertex of ${{\mathcal {J}}}$ which is the closest to *v*, and let $[u_0,v]$ be the geodesic string connecting $u_0$ ad *v*. Let $(u_0,v]:=[u_0,v]{\setminus } u_0$ be the shorter string. Assume $Supp({{\mathcal {T}}}{\setminus } v)^{<0}\not =\emptyset$. Then we can repeat the last part of the proof of Proposition 2, where $(u_0,v]$ plays the role of $\overline{{{\mathcal {I}}}}{\setminus } {{\mathcal {I}}}$ of the Proposition, and $\{u\in {{\mathcal {V}}}((u_0,v])\,:\, \delta _u\ge 3\}$ stays for ${{\mathcal {U}}}$ (still denoted by ${{\mathcal {U}}}$).

Indeed, the restriction of *C*(*l*) to $(u_0,v]$ has the next properties: the multiplicity of the cut at *v* is $b_v-a_v\le 0$, and the cut multiplicity at the other end, induced by $E_{u_0}$, is $m_{u_0}<0$. Since there exists at least one non-negative vertex-multiplicity, namely $m_v\ge 0$, there exists at least one connecting vertex $u_1\in {{\mathcal {U}}}$ whose cut-multiplicity is strict positive.

Then the sum of $z^{{{\mathcal {T}}}{\setminus }v}_{[j^*(l'_0)]}(\tilde{l}')$ over all the $\tilde{l}'$ cycles with this property is zero. Then, by induction over all vertices of ${{\mathcal {U}}}$, we obtain (22).

#### Step 2

Next, we assume that *v* is an end–vertex of ${{\mathcal {T}}}$, and the adjacent vertex *w* has $\delta _w\ge 3$. Then we reduce this case to the previous case 5.1.1: first we blow up the edge connecting *v* and *w*, then we apply  5.1.1, then we blow down the newly created vertex. We have to verify that the modified counting functions are stable with respect to these operations.

When we blow up the edge (*v*, *w*), then we create a new graph, denoted by ${\overline{{{\mathcal {T}}}}}$ with a newly created base element ${\bar{E}}_{new}\in L({\overline{{{\mathcal {T}}}}})$. There is a natural projection $\rho : L({\overline{{{\mathcal {T}}}}})\rightarrow L({{\mathcal {T}}})$, and $\rho ^*:L'({{\mathcal {T}}})\rightarrow L'({\overline{{{\mathcal {T}}}}})$, which satisfy the projection formula $(\rho ^*(l'),{\bar{l}})=(l',\rho ({\bar{l}}))$. In particular, $\rho ^*(E^*_u)={\bar{E}}^*_u$ (with natural notations). Hence if we denote by ${\overline{{{\mathcal {J}}}}}\subset {{\mathcal {V}}}({\overline{{{\mathcal {T}}}}})$ the same index set as ${{\mathcal {J}}}\subset {{\mathcal {V}}}({{\mathcal {T}}})$, then

23 $$\begin{aligned} z^{{\mathcal {T}}}_{[l'_0]}(l')=z^{{\overline{{{\mathcal {T}}}}}}_{[\rho ^*(l'_0)]}(\rho ^*(l')) \ \ \ \text{ and } \ \ \ q^{{\mathcal {T}}}_{[l'_0],{{\mathcal {J}}}}(l'_0)=q^{{\overline{{{\mathcal {T}}}}}}_{[\rho ^*(l'_0)],{\overline{{{\mathcal {J}}}}}} (\rho ^*(l'_0)). \end{aligned}$$

Next, ${\overline{{{\mathcal {T}}}}}{\setminus }v$ is the graph obtained from ${{\mathcal {T}}}{\setminus }v$ by blowing up the vertex *w*. By definition, $Z^{{\overline{{{\mathcal {T}}}}}{\setminus }v}$ has the shape $\rho ^*(Z^{{{\mathcal {T}}}{\setminus }v}({{\mathbf {t}}}))\cdot (1-{{\mathbf {t}}}^{{\bar{E}}^*_w})/ (1-{{\mathbf {t}}}^{{\bar{E}}^*_{new}})$, where $\rho ^*(\sum z(l'){{\mathbf {t}}}^{l'})=\sum z(l'){{\mathbf {t}}}^{\rho ^*(l')}$. Note that ${\bar{E}}^*_{new}={\bar{E}}^*_w+{\bar{E}}_{new}$, hence ${{\mathbf {t}}}^{{\bar{E}}^*_{new}}={{\mathbf {t}}}^{{\bar{E}}^*_w} \cdot t_{new}$. Therefore, when we restrict to the ${\overline{{{\mathcal {J}}}}}$ variables and we substitute $t_{new}=1$, the term $(1-{{\mathbf {t}}}^{{\bar{E}}^*_w})/(1-{{\mathbf {t}}}^{{\bar{E}}^*_{new}})$ becomes 1. Hence, the coefficients of the reduced series associated with ${{\mathcal {T}}}{\setminus }v$ and ${\overline{{{\mathcal {T}}}}}{\setminus }v$ can be compared as in the previous case (23).

#### Step 3

Now we consider the general situation. We prove (19) by induction over $\sum _{k\not =k'}|{{\mathcal {V}}}({{\mathcal {T}}}_{v,k})|$. If this sum is zero, then we apply case 5.1.2. Consider the general situation, and let $e\in \cup _{k\not =k'}{{\mathcal {V}}}({{\mathcal {T}}}_{v,k})$ be an end vertex of ${{\mathcal {T}}}$. Then, by cases 5.1.–5.1.2

24 $$\begin{aligned}&q^{{\mathcal {T}}}_{[l'_0],{{\mathcal {J}}}}\,(l'_0)-q^{{\mathcal {T}}}_{[l'_0],{{\mathcal {J}}}\cup e}\,(l'_0)= q^{{{\mathcal {T}}}{\setminus }e}_{[j^*(l'_0)],{{\mathcal {J}}}}\,(j^*(l'_0)), \end{aligned}$$

25 $$\begin{aligned}&q^{{\mathcal {T}}}_{[l'_0],{{\mathcal {J}}}\cup v}\,(l'_0)-q^{{\mathcal {T}}}_{[l'_0],{{\mathcal {J}}}\cup v\cup e}\,(l'_0)= q^{{{\mathcal {T}}}{\setminus }e}_{[j^*(l'_0)],{{\mathcal {J}}}\cup v}\,(j^*(l'_0)). \end{aligned}$$

Since $q^{{\mathcal {T}}}_{[l'_0],{{\mathcal {J}}}\cup e}\,(l'_0)=q^{{\mathcal {T}}}_{[l'_0],{{\mathcal {J}}}\cup v\cup e}\,(l'_0)$ by Proposition 2, and

26 $$\begin{aligned} q^{{{\mathcal {T}}}{\setminus }e}_{[j^*(l'_0)],{{\mathcal {J}}}}\,(j^*(l'_0))= q^{{{\mathcal {T}}}{\setminus }e}_{[j^*(l'_0)],{{\mathcal {J}}}\cup v}\,(j^*(l'_0)) +q^{{{\mathcal {T}}}_{v,k'}}_{[j^*_{v,k'}(l'_0)],{{\mathcal {J}}}}\,(j^*_{v,k'}(l'_0)) \end{aligned}$$

by the inductive step, the identity (19) follows. This ends the proof of Lemma 3 as well.

## The Proof of Theorem 6

### The proof

We will prove the identity (9) by induction on the cardinality $|{{\mathcal {I}}}|$ of ${{\mathcal {I}}}$.

#### Step 1

Assume that ${{\mathcal {I}}}$ contains exactly one element, say *v*. We will use the notations of 5.1, ${{\mathcal {T}}}{\setminus }v=\cup _k {{\mathcal {T}}}_{v,k}$. We have to prove

27 $$\begin{aligned} Q^{{\mathcal {T}}}_{[l'_0]}\,(l'_0)-Q^{{\mathcal {T}}}_{[l'_0],v}\,(l'_0)=\ \sum _k \ Q^{{{\mathcal {T}}}_{v,k}}_{[j^*_{v,k}(l'_0)]}(j^*_{v,k}(l'_0)). \end{aligned}$$

We rewrite this identity in terms of modified counting functions (as in 3.6). For the last sum we have to consider nonempty subsets ${{\mathcal {J}}}\subset {{\mathcal {V}}}{\setminus }v$. Hence, it is natural to organize the nonempty subsets of ${{\mathcal {V}}}$ as $\{v\}\cup \{{{\mathcal {J}}},{{\mathcal {J}}}\cup v\}_{{{\mathcal {J}}}\subset {{\mathcal {V}}}{\setminus }v,\ {{\mathcal {J}}}\not =\emptyset }$. The modified counting function associated with $\{v\}$ cancels with the second term $Q^{{\mathcal {T}}}_{[l'_0],v}\,(l'_0)$ of (27). Hence the left hand side of (27) becomes an alternating sum of expressions of type $q^{{\mathcal {T}}}_{[l'_0],{{\mathcal {J}}}}\,(l'_0)-q^{{\mathcal {T}}}_{[l'_0],{{\mathcal {J}}}\cup v}\,(l'_0)$. If ${{\mathcal {J}}}$ is not contained totally in only one ${{\mathcal {V}}}({{\mathcal {T}}}_{v,k})$ then $v\in {\overline{{{\mathcal {J}}}}}$, hence this expression is zero by Proposition 2. Therefore we can assume that there exists *k* such that ${{\mathcal {J}}}\subset {{\mathcal {V}}}({{\mathcal {T}}}_{v,k})$. Hence the expression (27) decomposes as a sum over *k* according to this inclusion. Then the needed identity is the subject of Lemma 3.

#### Step 2

Next, we take ${{\mathcal {I}}}$ with $|{{\mathcal {I}}}|\ge 2$, and we assume, by the inductive step, that the identity (9) is true for any graph ${{\mathcal {T}}}'$, any $h'\in H({{\mathcal {T}}}')$, and any subset ${{\mathcal {I}}}'\subset {{\mathcal {V}}}({{\mathcal {T}}}')$ with $|{{\mathcal {I}}}'|<|{{\mathcal {I}}}|$.

Recall that ${{\mathcal {T}}}{\setminus }{{\mathcal {I}}}=\cup _i {{\mathcal {T}}}_i$, and we wish to prove

28 $$\begin{aligned} Q^{{\mathcal {T}}}_{[l'_0]}\,(l'_0)=Q^{{\mathcal {T}}}_{[l'_0],{{\mathcal {I}}}}\,(l'_0)+\sum _i \ Q^{{{\mathcal {T}}}_i}_{[j^*_i(l'_0)]}(j^*_i(l'_0)). \end{aligned}$$

We choose some $v\in {{\mathcal {I}}}$, and we apply the inductive step for ${{\mathcal {T}}}{\setminus }v$ and ${{\mathcal {I}}}{\setminus }v$. Let ${{\mathcal {T}}}\setminus v=\cup _{k} {{\mathcal {T}}}_{v,k}$ be the connected components of ${{\mathcal {T}}}{\setminus }v$, and let $j^*_{v,k}$ the corresponding dual operators. Note that if ${{\mathcal {T}}}_i$ is contained in ${{\mathcal {T}}}_{v,k}$ then $j^*_{{{\mathcal {T}}}_i\subset {{\mathcal {T}}}_{v,k}}\circ j^*_{v,k}=j^*_i$. In particular, we get the following identity

29 $$\begin{aligned} \sum _k\ Q^{{{\mathcal {T}}}_{v,k}}_{[ j^*_{v,k}(l'_0)]}(j^*_{v,k}(l'_0))= \sum _k\ Q^{{{\mathcal {T}}}_{v,k}}_{[ j^*_{v,k}(l'_0)],{{\mathcal {I}}}\setminus v}(j^*_{v,k}(l'_0))+ \sum _i \ Q^{{{\mathcal {T}}}_i}_{[j^*_i(l'_0)]}(j^*_i(l'_0)).\nonumber \\ \end{aligned}$$

By induction this identity is valid for any $\tilde{l}'_0$ (instead of $j^*_{{{\mathcal {T}}}{\setminus }v\subset {{\mathcal {T}}}} (l'_0) =\oplus _k j^*_{v,k} (l'_0)$) from the lattice of ${{\mathcal {T}}}{\setminus }v$ (satisfying the required assumptions that its $E^*$–coefficients are sufficiently high). Hence it is true also for $\tilde{l}'_0=\oplus _k j^*_{v,k}(l'_0)$, and this identity, in this way, will be considered as a quasipolynomial identity in variable $l'_0\in L'$ (cf. discussion from  3.3). The difference between (28) and (29) is

30 $$\begin{aligned} Q^{{\mathcal {T}}}_{[l'_0]}\,(l'_0)-\sum _k\ Q^{{{\mathcal {T}}}_{v,k}}_{[ j^*_{v,k}(l'_0)]}(j^*_{v,k}(l'_0))= Q^{{\mathcal {T}}}_{[l'_0],{{\mathcal {I}}}}\,(l'_0)- \sum _k\ Q^{{{\mathcal {T}}}_{v,k}}_{[ j^*_{v,k}(l'_0)],{{\mathcal {I}}}\setminus v}(j^*_{v,k}(l'_0)).\nonumber \\ \end{aligned}$$

This identity (via induction) is equivalent with (9). But, for the left hand side of (30) one can apply (the already proved) (27). In particular, (9) is equivalent with

31 $$\begin{aligned} Q^{{\mathcal {T}}}_{[l'_0],{{\mathcal {I}}}}\,(l'_0)-Q^{{\mathcal {T}}}_{[l'_0],v}(l'_0)= \sum _k\ Q^{{{\mathcal {T}}}_{v,k}}_{[ j^*_{v,k}(l'_0)],{{\mathcal {I}}}\setminus v}(j^*_{v,k}(l'_0)). \end{aligned}$$

Next, we rewrite the identity (31) in terms of modified counting functions by the same principle as in 6.1.1. For the last sum we have to consider nonempty subsets ${{\mathcal {J}}}\subset {{\mathcal {I}}}{\setminus }v$, and we organize the nonempty subsets of ${{\mathcal {I}}}$ as $\{v\}\cup \{{{\mathcal {J}}},{{\mathcal {J}}}\cup v\}_{{{\mathcal {J}}}\subset {{\mathcal {I}}}{\setminus }v,\ {{\mathcal {J}}}\not =\emptyset }$. The modified counting function associated with $\{v\}$ cancels with the second term of (31). Hence the left hand side of (31) is again a combination of expressions of type $q^{{\mathcal {T}}}_{h,{{\mathcal {J}}}}(l'_0)-q^{{\mathcal {T}}}_{h,{{\mathcal {J}}}\cup v}(l'_0)$. If ${{\mathcal {J}}}$ is not contained totally in only one ${{\mathcal {V}}}({{\mathcal {T}}}_{v,k})$ then $v\in {\overline{{{\mathcal {J}}}}}$, hence this expression is zero by Proposition 2. Hence we can assume that there exists *k* such that ${{\mathcal {J}}}\subset {{\mathcal {V}}}({{\mathcal {T}}}_{v,k})$ and the expression (31) also decomposes as a sum over *k* according to this inclusion, and it becomes the statement of Lemma 3.

## The proof of the vanishing from Equation (7)

### Normal surface singularities. (For more details see [13, 20, 22, 27])

Assume that (*X*, *o*) is a complex analytic normal surface singularity, and let $\phi :\widetilde{X}\rightarrow X$ be a good resolution of (*X*, *o*). We denote the exceptional curve $\phi ^{-1}(0)$ by *E*, and let $\cup _vE_v$ be its irreducible components. Let ${{\mathcal {T}}}$ be the dual resolution graph associated with $\phi$ (which is automatically connected and negative definite). Then $\widetilde{X}$, as a smooth manifold, serves as the plumbing 4–manifold associated with ${{\mathcal {T}}}$, and $M=\partial \widetilde{X}$ is the plumbed 3-manifold (and also the ‘link’ of (*X*, *o*)). A resolution is minimal if there is no rational $E_v$ with $E_v^2=-1$. We will assume, similarly as above, that *M* is a rational homology sphere, and we will use the notations from the previous sections.

The group of analytic line bundles on $\widetilde{X}$ (up to isomorphism), $\mathrm{Pic}(\widetilde{X})$, appears in the exact sequence

32 $$\begin{aligned} 0\rightarrow \mathrm{Pic}^0(\widetilde{X})\rightarrow \mathrm{Pic}(\widetilde{X}){\mathop {\longrightarrow }\limits ^{c_1}} L'\rightarrow 0, \end{aligned}$$

where $c_1$ denotes the first Chern class of a line bundle. Furthermore, $\mathrm{Pic}^0(\widetilde{X})=H^1(\widetilde{X},{{\mathcal {O}}}_{\widetilde{X}})\simeq {\mathbb {C}}^{p_g}$, where $p_g$ is the *geometric genus* of (*X*, *o*). (*X*, *o*) is called *rational* if $p_g(X,o)=0$. Artin in [2, 3] characterised rationality topologically via their graphs. Such graphs are called ‘rational’.

The homomorphism $c_1$ admits a unique (group homomorphism) section $l'\mapsto {{\mathcal {O}}}(l')\in \mathrm{Pic}(\widetilde{X})$, such that $c_1({{\mathcal {O}}}(l'))=l'$, which extends the natural section $l\mapsto {{\mathcal {O}}}_{\widetilde{X}}(l)$ valid for integral cycles $l\in L$.

We say that for a singularity (*X*, *o*) and resolution $\phi$ the Seiberg–Witten Invariant Conjecture (SWIC) is valid (cf. [23, 28]) if for any $l'_0\in L'$ one has

33 $$\begin{aligned} Q^{{{\mathcal {T}}}} _{[l'_0]}(l'_0)+\mathfrak {sw}_{[-l_0']*\sigma _{can}} (M({{\mathcal {T}}}))+ \frac{(K + 2l_0')^2+ |{{\mathcal {V}}}|}{8}= -h^1(\widetilde{X},{{\mathcal {O}}}(-l_0')). \end{aligned}$$

For rational singularities (and for any resolution of them) the SWIC is valid, cf. [5, 22, 27].

The identity (33) connects topological invariants of (*X*, *o*) (left hand side) with analytic sheaf–cohomology invariants. There are two special regions for $l'_0$ when it simplifies. When $l'_0\in -K+{{\mathcal {S}}}'$ then by Generalized Grauert–Riemenschneider vanishing theorem $h^1(\widetilde{X},{{\mathcal {O}}}(-l'))=0$. Hence (33) identifies the counting function with the normalized Seiberg–Witten invariant (as in Theorem 1).

However, when ${{\mathcal {S}}}'\cap \{l'\,:\, l'\not \ge l'_0\}=\emptyset$, then $Q^{{{\mathcal {T}}}} _{[l'_0]}(l'_0)=0$ and the rank of the corresponding sheaf–cohomology is identified with the normalized Seiberg–Witten invariant. If both conditions are satisfied simultaneously then we obtain the vanishing of the normalized Seiberg–Witten invariants.

#### Cyclic quotient singularities [4]

Recall that (*X*, *o*) is called a cyclic quotient singularity if one of the following (equivalent) facts hold:

1. (*X*, *o*) is the quotient of $({\mathbb {C}}^2,0)$ by a cyclic group;
2. the graph ${{\mathcal {T}}}$ of the minimal resolution is a string;
3. there exists a finite map $p:(X,o)\rightarrow ({\mathbb {C}}^2,0)$, whose (reduced) discriminant (ramification locus) is included in the union of the two local coordinate axes of $({\mathbb {C}}^2,0)$.

Assume that (*X*, *o*) is a cyclic quotient singularity, and $\phi$ is its minimal resolution. Let $C_1$ and $C_2$ be two cuts of the end–vertices. Then there exists a finite projection $p:(X,o)\rightarrow ({\mathbb {C}}^2,0)$ such that the discriminant of *p* is included in $\cup _{i=1,2} p(\phi (C_i))$, and $\{p(\phi (C_i))\}_{i=1,2}$ might serve as local coordinate axes of $({\mathbb {C}}^2,0)$.

Cyclic quotient singularities are rational.

### The proof of Equation (7)

We start with a normal surface singularity (*X*, *o*), a fixed resolution $\phi$ with dual graph ${{\mathcal {T}}}$. We fix $h\in H$ and $r_h\in L'$ as above. We write ${{\mathcal {T}}}{\setminus }\mathcal {N}=\cup _i{{\mathcal {T}}}_i$. Then each ${{\mathcal {T}}}_i$ is a connected string, hence by contraction of the corresponding exceptional divisors indexed by ${{\mathcal {T}}}_i$ we obtain a cyclic quotient singularity $(X_i,0)$. For this singularity the SWIC is valid. This, for the line bundle ${{\mathcal {O}}}(-j^*_i(r_h))$, reads as

$$\begin{aligned}&Q^{{{\mathcal {T}}}_i} _{[j^*_i(r_h)]}(j^*_i(r_h))+\mathfrak {sw}_{[-j^*_i(r_h)]*\sigma _{can}} (M({{\mathcal {T}}}_i))\\&\quad + \frac{(K({{\mathcal {T}}}_i) + 2j^*_i(r_h))^2+ |{{\mathcal {V}}}({{\mathcal {T}}}_i)|}{8}= -h^1(\widetilde{X}({{\mathcal {T}}}_i),{{\mathcal {O}}}(-j^*_i(r_h))). \end{aligned}$$

Write ${{\mathcal {V}}}_i={{\mathcal {V}}}({{\mathcal {T}}}_i)$. Let $\{E_v\}_{v\in {{\mathcal {V}}}_i}$ be the exceptional curves indexed by ${{\mathcal {T}}}_i$, and let $\partial {{\mathcal {V}}}_i$ be those nodes of ${{\mathcal {T}}}$ which are adjacent with ${{\mathcal {V}}}_i$ (this set contains one or two elements). If $n\in \partial {{\mathcal {V}}}_i$ then let $w(n)\in {{\mathcal {V}}}_i$ adjacent with *n*. Then, if $r_h=\sum _{v\in {{\mathcal {V}}}} l'_vE_v$ then $j^*_i(r_h)=\sum _{v\in {{\mathcal {V}}}_i} l'_vE_v-\sum _{n\in \partial {{\mathcal {V}}}_i} l'_n E^*_{w(n)}$. Since each $l'_v\in [0,1)$, the cycle $j^*_i(r_h)$ has the form $r_{h_i}-l$, where $r_{h_i}$ is in the semi–open cube of $L'({{\mathcal {T}}}_i)$ and $l\in L({{\mathcal {T}}}_i)$, $l\ge 0$. In particular, $\{l'\in L'({{\mathcal {T}}}_i)\,:\, l'\not \ge j^*_i(r_h)\}\cap {{\mathcal {S}}}'({{\mathcal {T}}}_i)$ is empty and $Q^{{{\mathcal {T}}}_i}_{[j^*_i(r_h)]} (j^*_i(r_h))=0$.

Hence, the needed vanishing is equivalent with $h^1(\widetilde{X}({{\mathcal {T}}}_i), {{\mathcal {O}}}(-j^*_i(r_h)))=0$. Usually, by ‘standard’ vanishing theorems, see e.g. [18, Th.12.1], if in the resolution of a rational singularity $l'\in {{\mathcal {S}}}'$ then $h^1 ({{\mathcal {O}}}(-l'))=0$. However, in this case $j^*_i(r_h)\in {{\mathcal {S}}}'({{\mathcal {T}}}_i)$ is not necessarily true ($j^*_i(r_h)$ might have even negative *E*–coefficients), see Example 1, hence we need another (deeper) argument.

The proof relies on the structure of the *universal abelian covering* (UAC) of (*X*, *o*). Since $H=H_1(M,{\mathbb {Z}})$ is finite, the abelianization $\pi _1(M)\rightarrow H$ determines a regular covering of $M_a\rightarrow M$, and a normal surface singularity $(X_a,o)$ with link $M_a$, and a finite analytic covering $c:(X_a,o)\rightarrow (X,o)$ with ramification locus only at $o\in X$. It is called the UAC of (*X*, *o*) (see e.g. [22, 31, 33] and the references therein).

If $\phi :\widetilde{X}\rightarrow X$ is a good resolution of (*X*, *o*) then let $c':Z\rightarrow \widetilde{X}$ be the normalized pullback of *c* via $\phi$. The (reduced) branch locus of $c'$ is included in $\phi ^{-1}(o)=E$, and the Galois action of *H* extends to *Z* as well. Since *E* is a normal crossing divisor, the only singularities what *Z* might have are cyclic quotient singularities. Let $\psi :\widetilde{X}_a\rightarrow Z$ be a resolution of these singular points such that $(c'\circ \psi )^{-1}(E)$ is a normal crossing divisor. Set $\widetilde{c}:=c'\circ \psi$.34The point is that the cycles $r_h\in L'$ and the line bundles ${{\mathcal {O}}}(-r_h)\in \mathrm{Pic}(\widetilde{X})$ appear in a natural way via the UAC as follows: $\widetilde{c}_* {{\mathcal {O}}}_{\widetilde{X}_a}$ has an *H*–eigenspace decomposition [22, 23, 27, 33]

35 $$\begin{aligned} \widetilde{c}_* {{\mathcal {O}}}_{\widetilde{X}_a}=\oplus _{h\in H} \, {{\mathcal {O}}}(-r_h). \end{aligned}$$

Let $\widetilde{X}_i$ be a small neighbourhood of $\cup _{v\in {{\mathcal {V}}}_i}E_v$ in $\widetilde{X}$. It serves as the plumbed 4–manifold $\widetilde{X}({{\mathcal {T}}}_i)$ associated with ${{\mathcal {T}}}_i$, and the restriction of $\phi$ is a minimal resolution $\phi _i:\widetilde{X}_i\rightarrow X_i$ of the quotient singularity $(X_i,o)$. Consider the restriction ${{\mathcal {O}}}(-r_h)|_{\widetilde{X}_i}\in \mathrm{Pic}(\widetilde{X}_i)$. Its Chern class is $-j^*_i(r_h)\in L'({{\mathcal {T}}}_i)$. Since $p_g(X_i,o)=0$, by the exact sequence (32) ${{\mathcal {O}}}(-r_h)|_{\widetilde{X}_i}$ is determined by its Chern class, hence it is ${{\mathcal {O}}}(-j^*_i(r_h))$. Hence we need to prove that $h^1({{\mathcal {O}}}(-r_h)|_{\widetilde{X}_i})=0$.

Let $\widetilde{X}_{a,i}$ be $\widetilde{c}^{-1}(\widetilde{X}_i)$ in $\widetilde{X}_a$, and $\widetilde{c}_i:\widetilde{X}_{a,i}\rightarrow \widetilde{X}_i$ the restriction of $\widetilde{c}$. Then the *H* action preserves $\widetilde{X}_{a,i}$, and the eigenspace decomposition (35) is compatible with the restriction, hence ${{\mathcal {O}}}(-r_h)|_{\widetilde{X}_i}$ is a direct eigenspace summand (corresponding to *h*) of $(\widetilde{c}_{i})_*{{\mathcal {O}}}_{\widetilde{X}_{a,i}}$. Hence it is enough to prove that $h^1((\widetilde{c}_i)_*{{\mathcal {O}}}_{\widetilde{X}_{a,i}})=0$. Since $\psi$ resolves only cyclic quotient singularities, and $c'$ is finite, $R^1\widetilde{c}_*{{\mathcal {O}}}_{\widetilde{X}_a}=0$. Hence, by Leray spectral sequence, $h^1((\widetilde{c}_i)_*{{\mathcal {O}}}_{\widetilde{X}_{a,i}})= h^1({{\mathcal {O}}}_{\widetilde{X}_{a,i}})$. Thus, we need $h^1({{\mathcal {O}}}_{\widetilde{X}_{a,i}})=0$.

$\widetilde{X}_{a,i}$ has several (isomorphic) connected components. By construction, $\widetilde{c}_i$ is a regular covering off $(\cup _{v\in {{\mathcal {V}}}_i}E_v)\cup \cup _{n\in \partial {{\mathcal {V}}}_i}(E_n\cap \widetilde{X}_i)$. The disc(s) $C_n:=E_n\cap \widetilde{X}_i$ are/is cut(s) of ${{\mathcal {T}}}_i$ in $\widetilde{X}_i$ at the end–vertices. By the discussion from 7.1.1 there is a projection $p_i:(X_i,0)\rightarrow ({\mathbb {C}}^2,0)$, such that $p_i(\cup _n C_n)$ is included in the discriminant, which itself is included in the union of the coordinate axes. This $p_i$ composed with $\widetilde{c}_i$ provides a map $\widetilde{X}_{a,i}\rightarrow ({\mathbb {C}}^2,0)$ with discriminant included in the union of coordinate axes. Hence, each component of $\widetilde{X}_{a,i}$ is a resolution of a cyclic quotient singularity. Since cyclic quotient singularities are rational, $h^1({{\mathcal {O}}}_{\widetilde{X}_{a,i}})=0$.

#### Remark 3

From (35) one has $p_g(X_a,o)=\sum _{h\in H} h^1(\widetilde{X}, {{\mathcal {O}}}(-r_h))$.

#### Example 1

Let ${{\mathcal {T}}}$ be the left graph below, and at right we show the *E*–multiplicities of $r_h\in L'$.

para
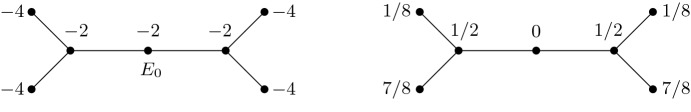


Let ${{\mathcal {T}}}_i$ be the subgraph consisting of the $(-2)$ vertex $E_0$ between the two nodes. Then $j^*_i(r_h)= -E_0^*=-E_0/2$. Hence, usually $j^*_i(r_h)$ is not even effective. The Chern class of ${{\mathcal {O}}}(-j^*_i(r_h))$ is $(E_0/2,E_0)=-1$. Since $h^1({{\mathcal {O}}}(-E_0+E_0/2))=0$ by Grauert–Riemenschneider type vanishing, we get $h^1({{\mathcal {O}}}(E_0/2))=h^1({{\mathcal {O}}}_{E_0}(E_0/2))= h^1({{\mathcal {O}}}_{{\mathbb {P}}^{1}}(-1))=0$.

#### Example 2

Consider the following graph ${{\mathcal {T}}}$ and the *E*–multiplicities of certain $r_h\in L'$. 
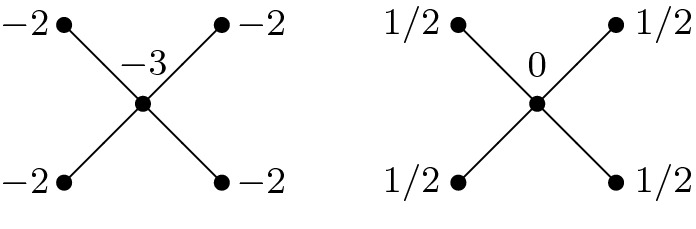


Let ${{\mathcal {I}}}$ be the union of the four $(-2)$–vertices, hence ${{\mathcal {T}}}_1= {{\mathcal {T}}}{\setminus }{{\mathcal {I}}}$ consists of the $(-3)$–vertex $E_0$. Then $j^*_1(r_h)= -2E_0^*=-2E_0/3$. Its representative $r_{h_1}$ is $E_0/3$, hence, usually, $j^*_i(r_h)\not =r_{h_i}$.

The Chern class of ${{\mathcal {O}}}(-j^*_1(r_h))$ is $(2E_0/3,E_0)=-2$. Hence, $h^1({{\mathcal {O}}}(2E_0/3))=h^1({{\mathcal {O}}}_{E_0}(2E_0/3))= h^1({{\mathcal {O}}}_{{\mathbb {P}}^{1}}(-2))=1$. Furthermore, $Q^{{{\mathcal {T}}}_i} _{[j^*_1(r_h)]}(j^*_1(r_h))=0$, thus

36 $$\begin{aligned} \mathfrak {sw}_{[-j^*_1(r_h)]*\sigma _{can}} (M({{\mathcal {T}}}_1))+ \frac{(K({{\mathcal {T}}}_1) + 2j^*_1(r_h))^2+ |{{\mathcal {V}}}({{\mathcal {T}}}_1)|}{8}= -1. \end{aligned}$$

## Application: ${{\mathcal {T}}}_i$ are rational

### Discussion

Consider the situation of Theorem 5 and assume that all ${{\mathcal {T}}}_i$ are rational (see [20] or Sect. 7). We will prove two ‘reduction formulae’, see Propositions 3 and 4.

If $h=0$ (hence $r_h=0$ too) then the SWIC is valid for the corresponding singularity, hence (33) applied for $l'_0=0$ reads as

37 $$\begin{aligned} \mathfrak {sw}_{\sigma _{can}}(M({{\mathcal {T}}}_i))+ \frac{K({{\mathcal {T}}}_i)^2+ |{{\mathcal {V}}}({{\mathcal {T}}}_i)|}{8}= 0. \end{aligned}$$

Hence, if each ${{\mathcal {T}}}_i$ is rational then

$$\begin{aligned} \mathfrak {sw}_{\sigma _{can}}(M)+\frac{K^2+|\mathcal {V}|}{8} = - pc ^{\pi _{{\mathcal {I}}}({{\mathcal {S}}}'_{\mathbb {R}})}(Z_0({\mathbf {t}}_{{\mathcal {I}}})), \end{aligned}$$

thus the ‘normalized Seiberg–Witten invariant associated with $\sigma _{can}$ can be computed as the periodic constant of the series reduced to the variables ${{\mathbf {t}}}_{{\mathcal {I}}}$.

In general, for arbitrary *h*, the vanishing (7) does not hold (even if ${{\mathcal {T}}}$ itself is rational), cf. Example 2. However, the contribution from ${{\mathcal {T}}}_i$ rational still can be simplified.

In order to state the results we need some preparation.

### Preparation

We fix a graph as in 2.1 and $h\in H$. Then there exists a unique representative $s_h\in {{\mathcal {S}}}'\subset L'$ of *h*, which is the unique minimal element (with respect to the partial ordering) of $\{s\in {{\mathcal {S}}}'\,:\, [s]=h\}$ [21, 22]. Usually $s_h\not =r_h$. Since $s_h\ge 0$, by the definition of $r_h$ we have $s_h-r_h=\varDelta _h\in L$, $\varDelta _h\ge 0$.

Note that for $s_h$ still $Q^{{\mathcal {T}}}_{h}(s_h)=0$ (since *Z* is supported on ${{\mathcal {S}}}'$, and in ${{\mathcal {S}}}'$ the representative $s_h$ is minimal in its class). Moreover, if ${{\mathcal {T}}}$ is rational, then for $s_h$ applies Lipman’s vanishing as well [18, Th.12.1], namely $h^1(\widetilde{X},{{\mathcal {O}}}(-l'))=0$ for any $l'\in {{\mathcal {S}}}'$. Thus, the SWIC for $l'_0=s_h$ reads as

38 $$\begin{aligned} \mathfrak {sw}_{-h*\sigma _{can}} (M({{\mathcal {T}}}))+ \frac{(K + 2s_h)^2+ |{{\mathcal {V}}}|}{8}= 0 \ \ (\text{ whenever } {{\mathcal {T}}} \text{ is } \text{ rational }). \end{aligned}$$

#### Remark 4

Since the lattice cohomology theory is a categorification of the normalized Seiberg–Witten invariants cf. [25], the above facts can also be reinterpreted by the lattice cohomological characterization of rational singularities (for more details see [24, 4.1]).

### First application

Consider the situation of Theorem 5 with ${{\mathcal {T}}}_i$ rational. Then (38) and Remark 1(2) imply

39 $$\begin{aligned} \mathfrak {sw}_{-[j^*_{i}(r_h)]*\sigma _{can,i}}(M_i)+\frac{(K(\mathcal {T}_i) + 2j^*_i(r_h))^2+ |\mathcal {V}({{\mathcal {T}}}_i)|}{8}=\chi (s_{h_i})-\chi (j^*_{i}(r_h)).\nonumber \\ \end{aligned}$$

Hence, if all ${{\mathcal {T}}}_i$ are rational then

#### Proposition 3

$$\begin{aligned} \mathfrak {sw}_{-h*\sigma _{can}}(M)+\frac{(K+2r_h)^2+|\mathcal {V}|}{8}&=\ - pc ^{\pi _{{\mathcal {I}}}({{\mathcal {S}}}'_{\mathbb {R}})}(Z_{h}^{{\mathcal {T}}}({\mathbf {t}}_{{\mathcal {I}}}))\\&\quad + \ \sum _{i} \Big ( \chi (s_{h_i})-\chi (j^*_{i}(r_h)) \Big ). \end{aligned}$$

### Second application

Consider again the situation of Theorem 5 with all ${{\mathcal {T}}}_i$ rational. Corollary 2 applied for $l'_0=s_h=r_h+\varDelta _h$ reads as

40 $$\begin{aligned} \begin{aligned}&\mathfrak {sw}_{-h*\sigma _{can}}(M)+\frac{(K+2s_h)^2+|\mathcal {V}|}{8} = \\&\sum _{i} \Big ( \mathfrak {sw}_{-[j^*_{i}(s_h)]*\sigma _{can,i}}(M_i)+\frac{(K(\mathcal {T}_i) + 2j^*_i(s_h))^2+ |\mathcal {V}({{\mathcal {T}}}_i)|}{8}\Big ) - \mathfrak {Q}^{{\mathcal {T}}}_{h,{{\mathcal {I}}}}\,(\varDelta _h). \end{aligned} \end{aligned}$$

The following fact follows directly from definitions, for details see [16, Lemma 8.4.2].

#### Lemma 4

$$j^{*}_i(s_{h}) = s_{[j^{*}_i(s_{h})]}$$ in $$L'_{\mathcal {T}_i}$$.

In particular, (38) applied for each ${{\mathcal {T}}}_i$ gives the vanishing of the $\sum _i$ in (40). Moreover, by Proposition 1 one has

$$\begin{aligned} \mathfrak {Q}^{{\mathcal {T}}}_{h,{{\mathcal {I}}}}\,(\varDelta _h)= ({{\mathbf {t}}}^{-\varDelta _h|_{{\mathcal {I}}}}Z_h^{{\mathcal {T}}}({{\mathbf {t}}}_{{\mathcal {I}}})|_{\not \ge 0})(\mathbf{1})+ \mathrm{pc}^{\pi _{{\mathcal {I}}}(S'_\mathbb {R})}({{\mathbf {t}}}^{-\varDelta _h|_{{\mathcal {I}}}}Z_h^{{\mathcal {T}}}({{\mathbf {t}}}_{{\mathcal {I}}})|_{\ge 0}). \end{aligned}$$

Hence we obtain the following reduction formula

#### Proposition 4

$$\begin{aligned}&\mathfrak {sw}_{-h*\sigma _{can}}(M)+\frac{(K+2s_h)^2+|\mathcal {V}|}{8}\\&\quad = - ({{\mathbf {t}}}^{-\varDelta _h|_{{\mathcal {I}}}}Z_h^{{\mathcal {T}}}({{\mathbf {t}}}_{{\mathcal {I}}})|_{\not \ge 0})(\mathbf{1})- \mathrm{pc}^{\pi _{{\mathcal {I}}}(S'_\mathbb {R})}({{\mathbf {t}}}^{-\varDelta _h|_{{\mathcal {I}}}}Z_h^{{\mathcal {T}}}({{\mathbf {t}}}_{{\mathcal {I}}})|_{\ge 0}). \end{aligned}$$

(By [14, (4.3.15)] on the right hand side one can replace $\varDelta _h$ by $s_h$, in this way the series will be ‘genuine’ series with integral exponents.)

### More examples and applications

1. The surgery formulae of this section generalize those surgery formulae, which reduce the lattice *L* to a lower rank lattice associated with ‘bad vertices’. We recall that a collection of vertices ${{\mathcal {I}}}$ of ${{\mathcal {V}}}$ is called ‘bad’ if by decreasing the decoration of these vertices on the graph we obtain a rational graph (cf. [15, 21]). Since the subgraph of a rational graph is rational, if ${{\mathcal {I}}}$ consists of ‘bad vertices’ then all components of ${{\mathcal {T}}}{\setminus }{{\mathcal {I}}}$ are rational. (Nevertheless, the converse is not true, see e.g. examples from [21, 8.2(5)].) In this sense our new surgery formula from Proposition 4 generalizes [15, Th. 5.3].
2. A special family of graph manifolds when ${{\mathcal {T}}}\setminus {{\mathcal {I}}}$ are all rational is provided by $S^3_{-d}(K)$, the $(-d)$-surgery along the connected sum $K=K_1\#\dots \#K_{\nu }\subset S^3$ of algebraic knots $K_{\ell }$. In this case there is a special vertex $v_+$ such that all the connected components of ${{\mathcal {T}}}{\setminus }v_+$ represent $S^3$. In this case $Z_0(t_{v_+})$ can be computed from the Alexander polynomials of the knots $K_{\ell }$, providing explicit formula for the Seiberg–Witten invariants in terms of these Alexander polynomials. For details see [5, 8.1] or [30, Th. 2.4.5].

## The case of numerically Gorenstein graphs

### Discussion

Recall that Corollary 2 assures that the counting function $Q^{{\mathcal {T}}}_{h,{{\mathcal {I}}}}(l_0')$ and its quasipolynomial $\mathfrak {Q}^{{\mathcal {T}}}_{h,{{\mathcal {I}}}}(l'_0-r_h)$ agree whenever all $a_v$ coefficients of $l_0'$ are sufficiently large. In general, for an arbitrary graph and *h* it is hard to determine a precise (and sharp) bound from which this equality holds. However, for numerically Gorenstein graphs and $h=0$ we determine such a bound. The presentation also shows the perfect parallelism of our ‘topological dualities’ with the (algebraic/analytic) Gorenstein (or Serre) dualities known in singularity theory or algebraic geometry.

### Definitions and notations

The connected negative definite graph ${{\mathcal {T}}}$ is called *numerically Gorenstein* if $K\in L$. In this section we assume that the graph is *minimal good* (that is, there exists no vertex *v* with $E_v^2=-1$ and $\delta _v\le 2$) and numerically Gorenstein, and we consider $h=0$ only. We denote the anticanonical cycle $-K$ by $Z_K$. Note that $Z_K=0$ if and only if ${{\mathcal {T}}}$ is ADE–graph (all decorations are $-2$), and in all other cases all the coefficients of $Z_K$ are strict positive [12, Prop. 2.1], [37, Cor. 2.8].

Recall that $\chi :L\rightarrow \mathbb {Z}$ was defined as $-(l,l-Z_K)/2$, hence the first trace of the duality/symmetry is $\chi (l)=\chi (Z_K-l)$.

Motivated by the theory of lattice cohomology (see e.g. [24, 25]) we consider for any ${{\mathcal {J}}}\subset {{\mathcal {V}}}$ lattice cubes $(l,{{\mathcal {J}}})$, of dimension $|{{\mathcal {J}}}|$, of the cubical decomposition given by $L\simeq \mathbb {Z}^{|{{\mathcal {V}}}|}\subset \mathbb {R}^{|{{\mathcal {V}}}|}$. The vertices of such a cube $(l,{{\mathcal {J}}})$ are $\{l+E_{{{\mathcal {J}}}'}\}_{{{\mathcal {J}}}'\subset {{\mathcal {J}}}}$ (where $E_{{{\mathcal {J}}}}=\sum _{v\in {{\mathcal {J}}}}E_v$). The weight of the cube $(l,{{\mathcal {J}}})$ is defined as $w(l, {{\mathcal {J}}}) = \max _{{{\mathcal {J}}}' \subset {{\mathcal {J}}}}\{\chi (l + E_{{{\mathcal {J}}}'})\}$.

For lattice points $a \le b$, $a,b \in L$, we define the rectangle *R*(*a*, *b*) by $\{x\in L\otimes \mathbb {R}\,:\, a \le x \le b\}$. Then the cube $(l,{{\mathcal {J}}})$ belongs to *R*(*a*, *b*) if $a\le l+E_{{{\mathcal {J}}}'}\le b$ for all its vertices.

The cycle $j_{{\mathcal {I}}}\pi _{{\mathcal {I}}}Z_K\in L$ has the same $E_v$–coefficient as $Z_K$ whenever $v\in {{\mathcal {I}}}$, otherwise it is zero. They define the rectangles $R({{\mathcal {I}}}):=R(j_{{\mathcal {I}}}\pi _{{\mathcal {I}}}Z_K, Z_K)$. E.g., $R(\emptyset ) =R(0,Z_K)$ and $R({{\mathcal {V}}})=R(Z_K,Z_K)$.

In the next discussion it is convenient to use the next abridged notation for any connected ${{\mathcal {T}}}$:

$$\begin{aligned} {\overline{\mathfrak {sw}}}({{\mathcal {T}}}) = -\mathfrak {sw}_{\sigma _{can}}(M({{\mathcal {T}}}))- (K^2+|\mathcal {V}|)/8. \end{aligned}$$

If ${{\mathcal {T}}}$ has several connected components, say ${{\mathcal {T}}}= \cup _{i}{{\mathcal {T}}}_i$, then we set $\overline{\mathfrak {sw}}({{\mathcal {T}}}) = \sum _{i }\overline{\mathfrak {sw}}({{\mathcal {T}}}_i)$.

The setup is as in Sect. 3: ${{\mathcal {I}}}\subset {{\mathcal {V}}}$ is non–empty, and ${{\mathcal {T}}}\setminus {{\mathcal {I}}}=\cup _i{{\mathcal {T}}}_i$. We write $Z^{{\mathcal {T}}}_0({{\mathbf {t}}})=\sum _{l\in L}z(l){{\mathbf {t}}}^l$.

We start with the following immediate consequence of (10) (use $j^*_i(K)=K({{\mathcal {T}}}_i)\in L'({{\mathcal {T}}}_i)$):

#### Proposition 5

(Topological duality of the quasipolynomial) The quasipolynomial $\mathfrak {Q}^{{\mathcal {T}}}_{0,{{\mathcal {I}}}}$ satisfies the symmetry $\mathfrak {Q}^{{\mathcal {T}}}_{0,{{\mathcal {I}}}}(l)=\mathfrak {Q}^{{\mathcal {T}}}_{0,{{\mathcal {I}}}}(Z_K-l)$, in particular $\mathrm{pc}^{\pi _{{\mathcal {I}}}({{\mathcal {S}}}'_\mathbb {R})}(Z_0({{\mathbf {t}}}_{{\mathcal {I}}}))= \mathfrak {Q}^{{\mathcal {T}}}_{0,{{\mathcal {I}}}}(0)=\mathfrak {Q}^{{\mathcal {T}}}_{0,{{\mathcal {I}}}}(Z_K)$.

### The main result and preparations for the proof

In the next formulae the rectangle $R(0,Z_K)$ will play a crucial role: basically we will express all our invariants as sums of weighted cubes of different faces of $R(0,Z_K)$.

The main result of this section is the following.

#### Theorem 7

Under the above assumptions $\mathfrak {Q}^{{\mathcal {T}}}_{0,{{\mathcal {I}}}}(Z_K)=Q^{{\mathcal {T}}}_{0,{{\mathcal {I}}}}(Z_K)$. In particular,

41 $$\begin{aligned} pc ^{\pi _{{\mathcal {I}}}({{\mathcal {S}}}'_{\mathbb {R}})}(Z_{0}({\mathbf {t}}_{{\mathcal {I}}})) = \mathfrak {Q}^{{\mathcal {T}}}_{0 ,{{\mathcal {I}}}}(Z_K) =Q^{{\mathcal {T}}}_{0,{{\mathcal {I}}}}(Z_K). \end{aligned}$$

The main advantage of (41) is that the the needed correction term in the surgery formulae, the usually hardly computable and more theoretical $pc ^{\pi _{{\mathcal {I}}}({{\mathcal {S}}}'_{\mathbb {R}})}(Z_{0}({\mathbf {t}}_{{\mathcal {I}}}))$, can be replaced by the directly computable $\sum _{l|_{{{\mathcal {I}}}}\not \ge Z_K|_{{{\mathcal {I}}}}}z(l)$. This shows that for such graphs the quasipolynomial $\mathfrak {Q}^{{\mathcal {T}}}_{0 ,{{\mathcal {I}}}}$ can be avoided.

#### Proof

First we recall some needed results. $\square$

#### Fact 1

[25, Th. 2.3.10] For any $l\in L$

42 $$\begin{aligned} z(l) = \sum _{{{\mathcal {J}}}\subset {{\mathcal {V}}}} (-1)^{|{{\mathcal {J}}}|+1} w(l, {{\mathcal {J}}}). \end{aligned}$$

#### Fact 2

[25, 15, §5.3] For any $b \in L$ which satisfies $b \ge Z_K$ one has

43 $$\begin{aligned} \overline{\mathfrak {sw}}({{\mathcal {T}}}) = \sum _{(l, {{\mathcal {J}}}) \subset R(0, b)} (-1)^{|{{\mathcal {J}}}|+1} w(l, {{\mathcal {J}}}). \end{aligned}$$

Furthermore, there is a combinatorial cancelation (‘contraction’) of cubes, which identifies

44 $$\begin{aligned}&Q^{{\mathcal {T}}}_{0,{{\mathcal {V}}}}(Z_K)=\sum _{l\not \ge Z_K} z(l)=\sum _{l\not \ge Z_K}\, \sum _{{{\mathcal {J}}}\subset {{\mathcal {V}}}} (-1)^{|{{\mathcal {J}}}|+1} w(l,{{\mathcal {J}}}) \ \ \nonumber \\&\quad \text{ with } \sum _{\begin{array}{c} (l,{{\mathcal {J}}})\subset R(\emptyset ),\\ {l\not = Z_K} \end{array}} (-1)^{|{{\mathcal {J}}}|+1} w(l,{{\mathcal {J}}}). \end{aligned}$$

In particular, the two identities combined provide

45 $$\begin{aligned} Q^{{\mathcal {T}}}_{0,{{\mathcal {V}}}}(Z_K)=w(Z_K,\emptyset )+\overline{\mathfrak {sw}}({{\mathcal {T}}})= \chi (Z_K)+\overline{\mathfrak {sw}}({{\mathcal {T}}}) =\overline{\mathfrak {sw}}({{\mathcal {T}}}). \end{aligned}$$

This result was stated for connected graphs, however it extends naturally to non–connected graphs as well by the additivity of $\chi$ and $Z_K$ over the connected components.

Note that (45) together with (6) and Proposition  5 imply Theorem 7 for ${{\mathcal {I}}}={{\mathcal {V}}}$, that is: $\mathfrak {Q}^{{\mathcal {T}}}_{0,{{\mathcal {V}}}}(Z_K)= \mathfrak {Q}^{{\mathcal {T}}}_{0,{{\mathcal {V}}}}(0)= \overline{\mathfrak {sw}}({{\mathcal {T}}})= Q^{{\mathcal {T}}}_{0,{{\mathcal {V}}}}(Z_K)$.

The very same combinatorial cancelation of (44) from [25, 15, §5.3, Lemma 5.3.3] (with completely identical proof) provides the following identity as well.

#### Fact 3

For any ${{\mathcal {I}}}\subset {{\mathcal {V}}}$, ${{\mathcal {I}}}\not =\emptyset$, one has

46 $$\begin{aligned} q^{{\mathcal {T}}}_{0,{{\mathcal {I}}}}(Z_K) = \sum _{(l,{{\mathcal {J}}})\subset R(\emptyset ){\setminus }\cup _{v\in {{\mathcal {I}}}} R(\{v\})} (-1)^{|{{\mathcal {J}}}|+1} w(l,{{\mathcal {J}}}). \end{aligned}$$

(In the sum those cubes do not appear which sit in the affine hyperplanes $l|_v=Z_K|_v$ for some $v\in {{\mathcal {I}}}$.)

### The proof

Let us introduce the function $\mathfrak {s}: \{\text{ set } \text{ of } \text{ graphs }\} \rightarrow \mathbb {Z}$, such that

47 $$\begin{aligned} \sum _{\mathcal {T}' \subset \mathcal {T}}\mathfrak {s}(\mathcal {T}') = \overline{\mathfrak {sw}}({{\mathcal {T}}}) \ \ \text{(with } \text{ the } \text{ convention } \mathfrak {s}(\emptyset )=\overline{\mathfrak {sw}}(\emptyset )=0\text{) }. \end{aligned}$$

By induction on $|\mathcal {V}({{\mathcal {T}}})|$ one shows that $\mathfrak {s}$ is uniquely defined by (47). Moreover, the property $\overline{\mathfrak {sw}}(\cup _{i=1}^r{{\mathcal {T}}}_i) = \sum _{i=1}^r\overline{\mathfrak {sw}}({{\mathcal {T}}}_i)$, valid for several connected components, transforms into $\mathfrak {s}(\cup _{i=1}^r{{\mathcal {T}}}_i)=0$ whenever $r\ge 2$. Furthermore, by combinatorial cancellation (or by Möbius invertion)

48 $$\begin{aligned} \mathfrak {s}(\mathcal {T}) = \sum _{{{\mathcal {I}}}\subset {{\mathcal {V}}}} \,(-1)^{|{{\mathcal {V}}}|- |{{\mathcal {I}}}|} \, \overline{\mathfrak {sw}}({{\mathcal {T}}}({{\mathcal {I}}})). \end{aligned}$$

#### Lemma 5

For any ${{\mathcal {I}}}\subset {{\mathcal {V}}}$ the following identity holds.

49 $$\begin{aligned} \mathfrak {s}({{\mathcal {T}}}({{\mathcal {I}}})) = \sum _{(l,{{\mathcal {J}}})\subset R({{\mathcal {V}}}\setminus {{\mathcal {I}}}){\setminus }\cup _{v\in {{\mathcal {I}}}} R({{\mathcal {V}}}{\setminus }({{\mathcal {I}}}\setminus v))} (-1)^{|{{\mathcal {J}}}|+1} w(l, {{\mathcal {J}}}). \end{aligned}$$

#### Proof

Since for any $l_{{\mathcal {I}}}\in L({{\mathcal {T}}}({{\mathcal {I}}}))$ one has $\chi _{{{\mathcal {T}}}({{\mathcal {I}}})}(l_{{\mathcal {I}}})= \chi (j_{{\mathcal {I}}}(l_{{\mathcal {I}}}))=\chi (Z_K-j_{{\mathcal {I}}}(l_{{\mathcal {I}}}))$, (43) implies

50 $$\begin{aligned} \sum _{(l, {{\mathcal {J}}}) \subset R({{\mathcal {V}}}{\setminus }{{\mathcal {I}}})} (-1)^{|{{\mathcal {J}}}|+1} w(l, {{\mathcal {J}}}) = \sum _{(l, {{\mathcal {J}}}) \subset R(0,Z_K|_{{\mathcal {I}}})} (-1)^{|{{\mathcal {J}}}|+1} w(l, {{\mathcal {J}}}), \end{aligned}$$

where the second sum is considered in the lattice $L({{\mathcal {T}}}({{\mathcal {I}}}))$. We claim that in this lattice $D:=Z_K|_{{\mathcal {I}}}- Z_K({{\mathcal {T}}}({{\mathcal {I}}}))\ge 0$. Indeed, by the two adjunction formulae, for any $v\in {{\mathcal {I}}}$, $(E_v, D)=-(E_v, Z_K|_{{{\mathcal {V}}}{\setminus }{{\mathcal {I}}}})\le 0$ (since $Z_K|_{{{\mathcal {V}}}{\setminus }{{\mathcal {I}}}}$ is effective), hence $D\in {{\mathcal {S}}}'({{\mathcal {T}}}({{\mathcal {I}}}))$, and $D\ge 0$. In particular, the right hand side of (50) via (43) is $\overline{\mathfrak {sw}}({{\mathcal {T}}}({{\mathcal {I}}}))$. This gives for any ${{\mathcal {I}}}\subset {{\mathcal {V}}}$

51 $$\begin{aligned} \overline{\mathfrak {sw}}({{\mathcal {T}}}({{\mathcal {I}}}))= \sum _{(l, {{\mathcal {J}}}) \subset R({{\mathcal {V}}}{\setminus }{{\mathcal {I}}})} (-1)^{|{{\mathcal {J}}}|+1} w(l, {{\mathcal {J}}}). \end{aligned}$$

By (48) and (51) and combinatorial cancelation:

$$\begin{aligned} \mathfrak {s}({{\mathcal {T}}}({{\mathcal {I}}}))&= \sum _{{{\mathcal {J}}}\subset {{\mathcal {I}}}} (-1)^{|{{\mathcal {I}}}|-|{{\mathcal {J}}}|}\cdot \overline{\mathfrak {sw}}({{\mathcal {T}}}({{\mathcal {J}}}))\\&= \sum _{{{\mathcal {J}}}\subset {{\mathcal {I}}}} (-1)^{|{{\mathcal {I}}}|-|{{\mathcal {J}}}|} \, \sum _{(l,{{\mathcal {K}}})\subset R({{\mathcal {V}}}{\setminus }{{\mathcal {J}}})} (-1)^{|{{\mathcal {K}}}|+1}w(l,{{\mathcal {K}}}), \end{aligned}$$

which equals the right hand side of (49). $\square$

Then, for any ${{\mathcal {J}}}\not =\emptyset$, (46) and Lemma 5 imply

$$\begin{aligned} \sum _{{{\mathcal {K}}}\supset {{\mathcal {J}}}} \mathfrak {s}({{\mathcal {T}}}({{\mathcal {K}}}))=q^{{\mathcal {T}}}_{0,{{\mathcal {J}}}}(Z_K). \end{aligned}$$

Therefore, for any ${{\mathcal {I}}}$,

$$\begin{aligned} \begin{aligned} Q^{{\mathcal {T}}}_{0,{{\mathcal {I}}}}(Z_K)&=\sum _{\emptyset \not ={{\mathcal {J}}}\subset {{\mathcal {I}}}} (-1)^{|{{\mathcal {J}}}|+1}q^{{\mathcal {T}}}_{0,{{\mathcal {J}}}}(Z_K)=\sum _{\emptyset \not ={{\mathcal {J}}}\subset {{\mathcal {I}}}} (-1)^{|{{\mathcal {J}}}|+1}\ \sum _{{{\mathcal {K}}}\supset {{\mathcal {J}}}} \mathfrak {s}({{\mathcal {T}}}({{\mathcal {K}}}))\\&=\sum _{{{\mathcal {K}}}\subset {{\mathcal {V}}}}\mathfrak {s}({{\mathcal {T}}}({{\mathcal {K}}}))\sum _{\emptyset \not = {{\mathcal {J}}}\subset {{\mathcal {K}}}\cap {{\mathcal {I}}}} (-1)^{|{{\mathcal {J}}}|+1} =\sum _{{{\mathcal {K}}}\subset {{\mathcal {V}}},\, {{\mathcal {K}}}\cap {{\mathcal {I}}}\not =\emptyset } \mathfrak {s}({{\mathcal {T}}}({{\mathcal {K}}})) \\&=\sum _{{{\mathcal {K}}}\subset {{\mathcal {V}}}} \mathfrak {s}({{\mathcal {T}}}({{\mathcal {K}}}))- \sum _{{{\mathcal {K}}}\subset {{\mathcal {V}}}{\setminus }{{\mathcal {I}}}} \mathfrak {s}({{\mathcal {T}}}({{\mathcal {K}}})) =\overline{\mathfrak {sw}}({{\mathcal {T}}})-\overline{\mathfrak {sw}}({{\mathcal {T}}}\setminus {{\mathcal {I}}})\\ {}&= pc ^{\pi _{{\mathcal {I}}}({{\mathcal {S}}}'_{\mathbb {R}})}(Z_{0}({\mathbf {t}}_{{\mathcal {I}}})). \end{aligned} \end{aligned}$$

This ends the proof of Theorem 7.

The formulae (43), (44), (46) and (51) provide explicit expressions for $\overline{\mathfrak {sw}}({{\mathcal {T}}})$, $Q^{{\mathcal {T}}}_{0,{{\mathcal {V}}}}(Z_K)$, $q^{{\mathcal {T}}}_{0,{{\mathcal {I}}}}(Z_K)$ and $\overline{\mathfrak {sw}}({{\mathcal {T}}}({{\mathcal {I}}}))$ in terms of weighted cubes of different faces of $R(0,Z_K)$.
